# Global, regional, and national estimates of the population at increased risk of severe COVID-19 due to underlying health conditions in 2020: a modelling study

**DOI:** 10.1016/S2214-109X(20)30264-3

**Published:** 2020-06-15

**Authors:** Andrew Clark, Mark Jit, Charlotte Warren-Gash, Bruce Guthrie, Harry H X Wang, Stewart W Mercer, Colin Sanderson, Martin McKee, Christopher Troeger, Kanyin L Ong, Francesco Checchi, Pablo Perel, Sarah Joseph, Hamish P Gibbs, Amitava Banerjee, Rosalind M Eggo, Centre for the Mathematical Modelling of Infectious Diseases COVID-19 working group, Emily S Nightingale, Kathleen O'Reilly, Thibaut Jombart, W John Edmunds, Alicia Rosello, Fiona Yueqian Sun, Katherine E Atkins, Nikos I Bosse, Samuel Clifford, Timothy W Russell, Arminder K Deol, Yang Liu, Simon R Procter, Quentin J Leclerc, Graham Medley, Gwen Knight, James D Munday, Adam J Kucharski, Carl A B Pearson, Petra Klepac, Kiesha Prem, Rein M G J Houben, Akira Endo, Stefan Flasche, Nicholas G Davies, Charlie Diamond, Kevin van Zandvoort, Sebastian Funk, Megan Auzenbergs, Eleanor M Rees, Damien C Tully, Jon C Emery, Billy J Quilty, Sam Abbott, Ch Julian Villabona-Arenas, Stéphane Hué, Joel Hellewell, Amy Gimma, Christopher I Jarvis

**Affiliations:** a Department of Health Services Research and Policy, London School of Hygiene & Tropical Medicine, London, UK; b Department of Infectious Disease Epidemiology, London School of Hygiene & Tropical Medicine, London, UK; c Department of Non-Communicable Disease Epidemiology, London School of Hygiene & Tropical Medicine, London, UK; d Centre for Population Health Sciences, Usher Institute, University of Edinburgh, Edinburgh, UK; e School of Public Health, Sun Yat-Sen University, Guangzhou, China; f Department for Health Metrics, University of Washington, Seattle, WA, USA; g Institute for Health Metrics and Evaluation, University of Washington, Seattle, WA, USA; h IAVI Human Immunology Laboratory, Imperial College London, London, UK; i Institute of Health Informatics, University College London, London, UK

## Abstract

**Background:**

The risk of severe COVID-19 if an individual becomes infected is known to be higher in older individuals and those with underlying health conditions. Understanding the number of individuals at increased risk of severe COVID-19 and how this varies between countries should inform the design of possible strategies to shield or vaccinate those at highest risk.

**Methods:**

We estimated the number of individuals at increased risk of severe disease (defined as those with at least one condition listed as “at increased risk of severe COVID-19” in current guidelines) by age (5-year age groups), sex, and country for 188 countries using prevalence data from the Global Burden of Diseases, Injuries, and Risk Factors Study (GBD) 2017 and UN population estimates for 2020. The list of underlying conditions relevant to COVID-19 was determined by mapping the conditions listed in GBD 2017 to those listed in guidelines published by WHO and public health agencies in the UK and the USA. We analysed data from two large multimorbidity studies to determine appropriate adjustment factors for clustering and multimorbidity. To help interpretation of the degree of risk among those at increased risk, we also estimated the number of individuals at high risk (defined as those that would require hospital admission if infected) using age-specific infection–hospitalisation ratios for COVID-19 estimated for mainland China and making adjustments to reflect country-specific differences in the prevalence of underlying conditions and frailty. We assumed males were twice at likely as females to be at high risk. We also calculated the number of individuals without an underlying condition that could be considered at increased risk because of their age, using minimum ages from 50 to 70 years. We generated uncertainty intervals (UIs) for our estimates by running low and high scenarios using the lower and upper 95% confidence limits for country population size, disease prevalences, multimorbidity fractions, and infection–hospitalisation ratios, and plausible low and high estimates for the degree of clustering, informed by multimorbidity studies.

**Findings:**

We estimated that 1·7 billion (UI 1·0–2·4) people, comprising 22% (UI 15–28) of the global population, have at least one underlying condition that puts them at increased risk of severe COVID-19 if infected (ranging from <5% of those younger than 20 years to >66% of those aged 70 years or older). We estimated that 349 million (186–787) people (4% [3–9] of the global population) are at high risk of severe COVID-19 and would require hospital admission if infected (ranging from <1% of those younger than 20 years to approximately 20% of those aged 70 years or older). We estimated 6% (3–12) of males to be at high risk compared with 3% (2–7) of females. The share of the population at increased risk was highest in countries with older populations, African countries with high HIV/AIDS prevalence, and small island nations with high diabetes prevalence. Estimates of the number of individuals at increased risk were most sensitive to the prevalence of chronic kidney disease, diabetes, cardiovascular disease, and chronic respiratory disease.

**Interpretation:**

About one in five individuals worldwide could be at increased risk of severe COVID-19, should they become infected, due to underlying health conditions, but this risk varies considerably by age. Our estimates are uncertain, and focus on underlying conditions rather than other risk factors such as ethnicity, socioeconomic deprivation, and obesity, but provide a starting point for considering the number of individuals that might need to be shielded or vaccinated as the global pandemic unfolds.

**Funding:**

UK Department for International Development, Wellcome Trust, Health Data Research UK, Medical Research Council, and National Institute for Health Research.

## Introduction

Emerging evidence from China, Europe, and the USA has shown a consistently higher risk of severe COVID-19 in older individuals and those with underlying health conditions.^1^, ^2^, ^3^ Severe disease is defined by WHO as “a patient with severe acute respiratory illness (fever and at least one sign/symptom of respiratory disease, e.g., cough, shortness of breath; AND requiring hospitalization)”.^4^, ^5^ In a recent report from the USA, underlying conditions were reported in 71% (732/1037) of individuals admitted to hospital with COVID-19 and in 94% (173/184) of deaths.^1^ WHO, along with public health agencies in countries such as the UK and the USA, have issued guidelines on who is considered to be at increased risk of severe COVID-19.^6^, ^7^, ^8^ This includes individuals with cardiovascular disease, chronic kidney disease, diabetes, chronic respiratory disease, and a range of other chronic conditions. Such conditions increase the risk of needing hospital-based treatment such as oxygen supplementation. A large proportion of the additional health-care burden of COVID-19 epidemics is likely to result from infection of those with underlying conditions.

**Research in context**

**Evidence before this study**

As the COVID-19 pandemic evolves, countries are considering policies to protect those at increased risk of severe disease. This can involve policies to suppress transmission in the wider population, vaccination (if a vaccine becomes available), or so-called shielding—ie, specific measures to protect those at increased risk by minimising interactions between individuals at increased risk and others. Guidelines on who is currently believed to be at increased risk of severe COVID-19 have been published online by WHO and public health agencies in the UK and the USA. We searched PubMed using the terms “risk factors” AND “COVID-19” without language restrictions, from database inception until April 5, 2020, and identified 62 studies published between Feb 15 and March 20, 2020. Evidence from China, Europe, and the USA indicates that older individuals, males, and those with underlying conditions such as cardiovascular disease and diabetes are at increased risk of severe COVID-19 and death. At the time of the search, none of the studies identified aimed to quantify the number of individuals at increased risk due to underlying health conditions.

**Added value of this study**

This study combines evidence from large international databases and new analyses of large multimorbidity studies to inform policy makers about the number of individuals that might be at increased risk or high risk of severe COVID-19 in different countries. We developed a tool for rapid assessments of the number and percentage of country populations that would need to be targeted under different policies to protect those at increased risk.

**Implications of all the available evidence**

Estimating the number of people at increased risk of severe COVID-19 is crucial to help countries to design more effective interventions to protect vulnerable individuals and reduce pressure on health systems. This information can also inform a broader assessment of the health, social, and economic implications of shielding various groups.

Identifying at-risk populations is important not only for making projections of the probable health burden in countries,^9^, ^10^ but also for the design of effective strategies that aim to reduce the risk of transmission to people in target groups. This is sometimes termed shielding, defined by WHO^11^ as “measures to protect vulnerable persons at increased risk of severe disease from COVID-19...or increased risk of infection”—eg, by minimising interactions between individuals at increased risk and others. The specific definition of shielding can vary from one country to the next, but in general it has the potential to reduce mortality in susceptible groups (direct benefits), while at the same time mitigating the expected surge in demand for hospital beds (indirect benefits). However, trying to shield an excessive proportion of a population can strain country resources and reduce the overall effectiveness of shielding. A detailed assessment of the number of at-risk individuals can inform possible shielding strategies. If a vaccine becomes available in the future, it could also be used to inform the number of people with underlying conditions who would need to be vaccinated.

The aim of this analysis is to provide global, regional, and national estimates of the number of individuals at increased risk of severe COVID-19 as a result of their underlying medical conditions during 2020.

## Methods

### Prevalence of underlying health conditions

We mapped the conditions listed in the Global Burden of Diseases, Risk Factors, and Injuries Study (GBD)^12^ to lists of conditions associated with increased risk of severe COVID-19 from guidelines published by WHO and agencies in the UK and USA.^6^, ^7^, ^8^ The mapping was completed by a clinical epidemiologist (CW-G). Prevalence estimates were extracted by age, sex, and country and grouped into the following 11 categories: (1) cardiovascular disease, including cardiovascular disease caused by hypertension; (2) chronic kidney disease, including chronic kidney disease caused by hypertension; (3) chronic respiratory disease; (4) chronic liver disease; (5) diabetes; (6) cancers with direct immunosuppression; (7) cancers without direct immunosuppression, but with possible immunosuppression caused by treatment; (8) HIV/AIDS; (9) tuberculosis (excluding latent infections); (10) chronic neurological disorders; and (11) sickle cell disorders. A full list of GBD causes included in these categories is shown in the appendix (p 2).

We estimated the current number of individuals with underlying conditions making them at risk of severe COVID-19 by age (5-year age groups), sex, and country for 188 countries. Data on the prevalence of underlying conditions were extracted by age, sex, and country from GBD 2017 using the GBD results tool and combined with UN mid-year population estimates for 2020 for the 188 countries available.^13^ Countries were grouped by UN geographical regions.^14^ For this part of the analysis, older individuals without underlying conditions were not considered to be at increased risk.

Asthma is relatively common, and only moderate to severe asthma is listed as an increased-risk condition in guidelines in the USA, so we modified GBD estimates of asthma to account only for moderate to severe cases (defined as British Thoracic Society Steps 4, 5, and 6).^15^ Using evidence from the UK,^16^ we assumed these cases accounted for 15% of total asthma cases younger than 5 years, 17% aged 5–19 years, 23% aged 20–54 years, and 43% in those aged 55 years or older.

For HIV/AIDS, we included all populations, including those on antiretroviral therapy (ART). We did a sensitivity analysis to determine how estimates would change if we removed individuals using ART, in the case no additional risk of severe COVID-19 was found in individuals on ART. We used WHO national estimates for ART coverage among those living with HIV/AIDS.^17^

### Estimating individuals at increased risk

We estimated the percentage of country populations at increased risk of severe COVID-19 (those with at least one underlying condition listed as “at increased risk” in guidelines^6^, ^7^, ^8^) with and without age standardisation. GBD provides prevalence estimates for each disease separately, but does not provide the prevalence of people who have more than one disease. Diseases can cluster—for example, if they are causally related. To address this, we first calculated *e*, the expected proportion of individuals with at least one COVID-19-related condition—assuming no clustering and that the prevalences involved are independent (eg, the fact that someone has diabetes does not affect their risk of getting cancer)—as 1 minus the probability of not having a condition in any of the 11 categories c_i_: 1 − [1 − p(c_1_)] × [1 − p(c_2_)] × [1 − p(c_3_)] × … × [1 − p(c_11_)].

We then estimated the proportion *P* who have at least one underlying condition as *P* = *e* × *r*, where *r* is the ratio between the observed and expected percentage of individuals with at least one condition. We based *r* on evidence from large cross-sectional multimorbidity studies in Scotland^18^ and southern China^19^ (appendix pp 3–4).

### Adjustment for multimorbidity

In addition to providing estimates for *r*, the studies^18^, ^19^ in Scotland and southern China were used to calculate the multimorbidity fraction—ie, the proportion of individuals with at least two underlying conditions—among those with at least one condition, by age group and sex. For these calculations, we used disease categories in the two studies that matched as closely as possible to the COVID-19-relevant categories defined in our analysis. In both studies, this included cardiovascular disease (defined as the presence of one or more of coronary heart disease, hypertension, cerebrovascular disease, peripheral arterial disease, heart failure, or atrial fibrillation), chronic neurological disorders (defined as one or more of dementia, multiple sclerosis, and Parkinson's disease), and chronic respiratory disease (defined as one or both of chronic obstructive pulmonary disease and bronchiectasis). The remaining COVID-19-related conditions listed previously were counted separately. GBD provides separate estimates for hypertensive heart disease and chronic kidney disease due to hypertension, but it was not possible to make this distinction in the multimorbidity datasets, so all hypertension was included in the cardiovascular disease category.

Using data from both studies, we calculated pooled estimates of the ratio *r* and the multimorbidity fraction by age and sex (appendix pp 3–4) and applied these pooled estimates to all countries in the analysis.

### Inclusion of older individuals without underlying conditions

Some countries have also considered older age as a proxy for frailty and thus increased risk of severe COVID-19. Although frailty correlates much more closely with mortality than chronological age, there is a well established non-linear association between increasing age and frailty.^20^ We therefore calculated the number of individuals without an underlying condition that could be considered at increased risk because of their age, using age thresholds ranging from 50 to 70 years. All age thresholds were evaluated in all regions. To calculate the total number at increased risk for different age thresholds, we added the number of older individuals without underlying conditions to our previous estimates of the number of individuals with at least one underlying condition.

### Estimating individuals at high risk

To aid interpretation of the degree of risk among individuals at increased risk, we also estimated the number of individuals at high risk, defined as those that would require hospital admission if infected, calculated using previously estimated age-specific infection–hospitalisation ratios (IHRs) for COVID-19. This risk group includes infections and severe cases in the wider population (irrespective of whether they had underlying conditions). Thus the high-risk group is not a precise subset of the increased-risk group because it includes some severe cases without underlying conditions. To estimate numbers at high risk, we applied country-level UN estimates of the number of individuals alive in each 5-year age group^13^ to age-specific IHRs recently estimated for mainland China by Verity and colleagues.^21^ We made two adjustments to account for differences between IHRs in China and other countries (appendix pp 5–6). The first was designed to capture the effect on IHRs of national variations in prevalence mix compared with China. For each 5-year age group and sex, the prevalence rates for each underlying condition were multiplied by their respective relative risks (RRs) for hospitalisation of 3·0 for chronic kidney disease, diabetes, and cardiovascular disease and of 1·5 for the eight other conditions. The totals were then summed across all 11 conditions and added to the proportion of individuals without underlying conditions, to create a risk score for each 5-year age group. IHRs were then multiplied by the ratio of the risk score for the country of interest and China. The RRs used were based on studies that allowed comparison of hospitalised and non-hospitalised people with COVID-19 and were assumed to be the same for every country (appendix pp 5–10). The second was to adjust for infections in given age groups being more severe in higher mortality settings, using differences in age-specific life expectancy as a proxy, multiplying the IHR for each country by the ratio of age-specific life expectancy between China and that country.

Sex is not included in current guidelines but studies have shown an association between male sex and hospital admission. We therefore assumed males were twice as likely to be at high risk in all age groups (appendix p 8).^22^, ^23^, ^24^

### Uncertainty

For numbers at increased risk, we generated uncertainty intervals (UIs) by running low and high scenarios using the lower and upper 95% confidence limits for age-specific and sex-specific country population size, disease prevalences, and multimorbidity fractions. The UN population estimates for 2020 are not provided with 95% CIs, so we generated 95% CIs that were consistent with the 95% UIs around the GBD 2017 population estimates.^25^ Within our low and high scenarios, we also varied *r*, the ratio between the observed and expected percentage of individuals with at least one condition, in a range of 0·7 to 1·0, informed by the multimorbidity studies.^18^, ^19^ We ran a jack-knife analysis to show the influence of each underlying condition on the results by excluding each of the conditions, one at a time.

For estimates of numbers at high risk, we generated UIs using the low and high credible interval values of the IHRs reported by Verity and colleagues^21^ and the low and high 95% confidence limits for the country population size. We also ran several scenarios to assess the influence of our country-specific adjustments for underlying conditions and age-based frailty, and the RRs associated with each condition, ranging RRs from 1 to 10 for each individual condition (appendix pp 11–12).

All analyses are provided in an Excel spreadsheet tool. The tool can be used for rapid assessment and visualisation of the estimated number and percentage of country populations targeted under different shielding policies.

### Role of the funding source

The funder of the study had no role in study design, data collection, data analysis, data interpretation, or writing of the report. The corresponding author had full access to all of the data and the final responsibility to submit for publication.

## Results

**Figure 1:**
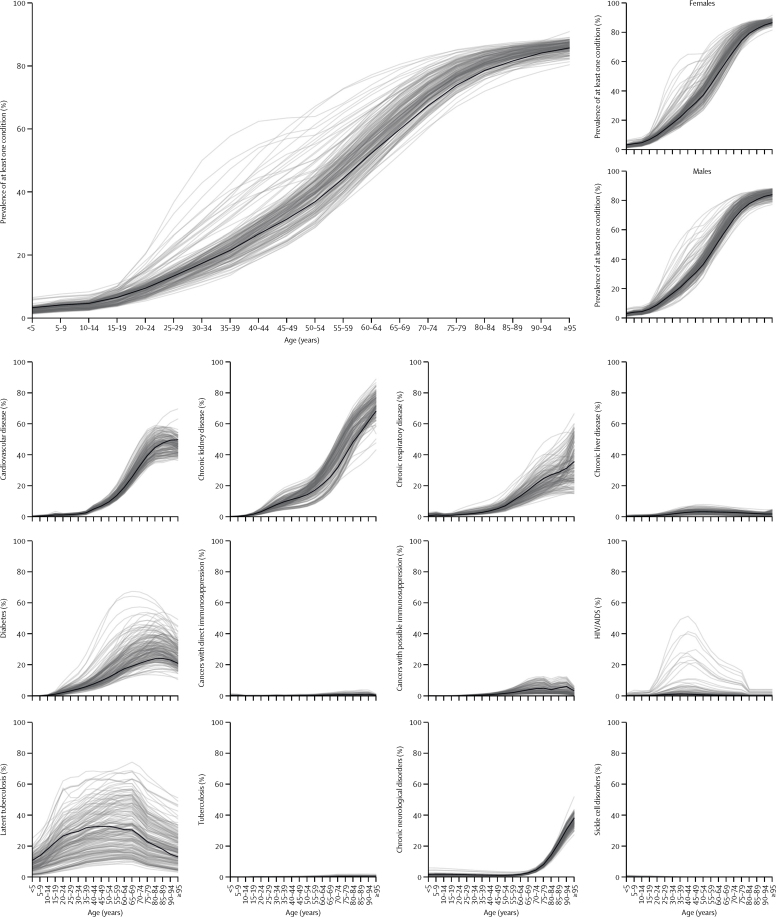
Global proportion of individuals with at least one underlying condition, by age and sex, and global prevalence of each underlying condition by age Grey lines represent individual countries and show variation around the global estimates (black lines). We excluded latent tuberculosis from our analysis but include it here to show the extent of overall tuberculosis that was excluded.

We estimated that 1·7 billion (UI 1·0–2·4) individuals, comprising 22% (15–28) of the global population, have at least one underlying condition that could increase their risk of severe COVID-19 (table 1; table 2; appendix pp 13–14). This value does not include older individuals without underlying conditions. The prevalence of one or more conditions was approximately 10% by age 25 years, 33% by 50 years, and 66% by 70 years, and similar for males and females (figure 1). The most prevalent conditions in those aged 50 years or older were chronic kidney disease, cardiovascular disease, chronic respiratory disease, and diabetes. These were also the most influential when conditions were removed from the analysis one at time (appendix p 15).

**Table 1:** Number of individuals in millions at increased risk of severe COVID-19 illness by age, number of conditions, region, and age threshold

		**Africa (n=1338·8 million)**	**Asia (n=4632·9 million)**	**Europe (n=747·1 million)**	**Latin America and the Caribbean (n=652·2 million)**	**Northern America (n=368·7 million)**	**Oceania (n=41·9 million)**	**Global (n=7781·7 million)**
**Population by number of conditions**								
No conditions								
	<15 years	519·2 (39%)	1042·4 (22%)	117·2 (16%)	151·6 (23%)	65·0 (18%)	9·5 (23%)	1905·0 (24%)
	15–49 years	533·6 (40%)	1985·9 (43%)	274·3 (37%)	291·6 (45%)	146·6 (40%)	16·7 (40%)	3248·8 (42%)
	50–54 years	24·8 (2%)	184·3 (4%)	33·4 (4%)	22·9 (4%)	14·5 (4%)	1·5 (4%)	281·5 (4%)
	55–59 years	17·6 (1%)	136·9 (3%)	29·4 (4%)	18·0 (3%)	12·9 (3%)	1·3 (3%)	216·1 (3%)
	60–64 years	11·6 (<1%)	95·8 (2%)	22·6 (3%)	12·5 (2%)	10·0 (3%)	1·0 (2%)	153·6 (2%)
	65–69 years	7·0 (<1%)	69·5 (1%)	16·0 (2%)	8·3 (1%)	6·7 (2%)	0·7 (2%)	108·2 (1%)
	≥70 years	6·6 (<1%)	73·8 (2%)	23·2 (3%)	9·9 (2%)	8·5 (2%)	1·0 (2%)	122·9 (2%)
	All ages	1120·5 (84%)	3588·5 (77%)	516·1 (69%)	514·8 (79%)	264·4 (72%)	31·8 (76%)	6036·0 (78%)
One condition only								
	<15 years	19·9 (1%)	43·5 (<1%)	2·7 (<1%)	4·0 (<1%)	1·6 (<1%)	0·3 (<1%)	71·9 (<1%)
	15–49 years	100·8 (8%)	367·6 (8%)	49·1 (7%)	45·1 (7%)	19·7 (5%)	2·9 (7%)	585·2 (8%)
	50–54 years	13·4 (1%)	82·3 (2%)	14·1 (2%)	10·3 (2%)	6·6 (2%)	0·6 (1%)	127·3 (2%)
	55–59 years	12·2 (<1%)	77·4 (2%)	17·3 (2%)	10·4 (2%)	8·4 (2%)	0·7 (2%)	126·4 (2%)
	60–64 years	10·5 (<1%)	68·6 (1%)	18·3 (2%)	9·5 (1%)	9·2 (3%)	0·7 (2%)	116·8 (2%)
	65–69 years	8·3 (<1%)	61·2 (1%)	17·5 (2%)	8·1 (1%)	8·6 (2%)	0·6 (2%)	104·3 (1%)
	≥70 years	11·3 (<1%)	92·1 (2%)	39·4 (5%)	14·5 (2%)	17·4 (5%)	1·4 (3%)	176·1 (2%)
	All ages	176·4 (13%)	792·6 (17%)	158·4 (21%)	102·0 (16%)	71·6 (19%)	7·3 (17%)	1308·2 (17%)
Multiple (two or more) conditions								
	<15 years	1·3 (<1%)	2·8 (<1%)	0·2 (<1%)	0·3 (<1%)	0·1 (<1%)	0·0 (<1%)	4·6 (<1%)
	15–49 years	14·1 (1%)	55·1 (1%)	7·9 (1%)	6·9 (1%)	3·2 (<1%)	0·4 (1%)	87·7 (1%)
	50–54 years	3·8 (<1%)	23·3 (<1%)	4·0 (<1%)	2·9 (<1%)	1·9 (<1%)	0·2 (<1%)	36·0 (<1%)
	55–59 years	4·3 (<1%)	27·1 (<1%)	6·0 (<1%)	3·7 (<1%)	3·0 (<1%)	0·2 (<1%)	44·3 (<1%)
	60–64 years	4·6 (<1%)	29·8 (<1%)	8·0 (1%)	4·1 (<1%)	4·0 (1%)	0·3 (<1%)	50·8 (<1%)
	65–69 years	4·5 (<1%)	33·1 (<1%)	9·5 (1%)	4·4 (<1%)	4·6 (1%)	0·3 (<1%)	56·4 (<1%)
	≥70 years	9·3 (<1%)	80·6 (2%)	37·1 (5%)	13·2 (2%)	16·0 (4%)	1·3 (3%)	157·6 (2%)
	All ages	41·9 (3%)	251·8 (5%)	72·7 (10%)	35·5 (5%)	32·8 (9%)	2·8 (7%)	437·4 (6%)
**Population at increased risk of severe COVID-19**								
People with at least one condition (all ages), assuming no age-based threshold		218·3 (16%)	1044·4 (23%)	231·0 (31%)	137·4 (21%)	104·4 (28%)	10·1 (24%)	1745·6 (22%)
Older people with no conditions*								
	≥50 years	67·7 (5%)	560·3 (12%)	124·6 (17%)	71·5 (11%)	52·7 (14%)	5·5 (13%)	882·3 (11%)
	≥55 years	42·9 (3%)	375·9 (8%)	91·2 (12%)	48·7 (7%)	38·2 (10%)	4·0 (9%)	600·8 (8%)
	≥60 years	25·2 (2%)	239·1 (5%)	61·8 (8%)	30·7 (5%)	25·3 (7%)	2·6 (6%)	384·8 (5%)
	≥65 years	13·6 (1%)	143·2 (3%)	39·3 (5%)	18·2 (3%)	15·3 (4%)	1·6 (4%)	231·2 (3%)
	≥70 years	6·6 (<1%)	73·8 (2%)	23·2 (3%)	9·9 (2%)	8·5 (2%)	1·0 (2%)	122·9 (2%)
People with at least one condition plus older people with no conditions*								
	≥50 years	286·0 (21%)	1604·7 (35%)	355·7 (48%)	209·0 (32%)	157·1 (43%)	15·6 (37%)	2627·9 (34%)
	≥55 years	261·2 (20%)	1420·4 (31%)	322·3 (43%)	186·1 (29%)	142·6 (39%)	14·0 (34%)	2346·5 (30%)
	≥60 years	243·5 (18%)	1283·5 (28%)	292·8 (39%)	168·1 (26%)	129·7 (35%)	12·7 (30%)	2130·4 (27%)
	≥65 years	231·9 (17%)	1187·7 (26%)	270·3 (36%)	155·6 (24%)	119·7 (32%)	11·7 (28%)	1976·8 (25%)
	≥70 years	224·9 (17%)	1118·2 (24%)	254·2 (34%)	147·3 (23%)	112·9 (31%)	11·0 (26%)	1868·6 (24%)

Data are number of individuals in millions (percentage of total population of the region).

* Older people with no conditions could be considered at increased risk by virtue of age alone.

**Table 2:** Global number and percentage of individuals at increased risk and high risk of severe COVID-19 by age and sex

	**Increased risk**			**High risk**		
	Number in millions (UI*)	Percentage (UI*)	Number per population	Number in millions (UI*)	Percentage (UI*)	Number per population
**Both sexes combined**						
All ages	1746 (1032–2398)	22% (15–28)	1/4·5	349 (186–787)	4% (3–9)	1/22·3
<20 years	116 (50–167)	4% (2–6)	1/22·4	3 (1–7)	0% (0–0)	1/916·4
20–29 years	134 (70–198)	11% (7–15)	1/8·9	16 (9–37)	1% (1–3)	1/73·6
30–39 years	220 (122–320)	19% (12–25)	1/5·2	38 (20–87)	3% (2–7)	1/30·0
40–49 years	279 (163–392)	29% (19–36)	1/3·5	50 (27–114)	5% (3–11)	1/19·2
50–54 years	163 (98–225)	37% (25–46)	1/2·7	34 (18–76)	8% (4–15)	1/13·2
55–59 years	171 (104–230)	44% (30–54)	1/2·3	41 (22–92)	11% (6–21)	1/9·5
60–64 years	168 (104–224)	52% (36–63)	1/1·9	39 (21–87)	12% (7–25)	1/8·3
65–69 years	161 (101–212)	60% (42–71)	1/1·7	41 (22–92)	15% (9–31)	1/6·6
≥70 years	334 (219–429)	73% (53–85)	1/1·4	87 (47–196)	19% (11–39)	1/5·2
**Females**						
All ages	907 (538–1242)	24% (16–29)	1/4·3	123 (66–278)	3% (2–7)	1/31·3
<20 years	58 (26–83)	5% (2–6)	1/21·7	1 (0–2)	0% (0–0)	1/1390·6
20–29 years	67 (35–99)	12% (7–15)	1/8·5	5 (3–12)	1% (1–2)	1/111·3
30–39 years	111 (62–161)	20% (12–26)	1/5·1	12 (7–28)	2% (1–5)	1/45·1
40–49 years	141 (82–198)	29% (19–37)	1/3·4	17 (9–38)	3% (2–7)	1/28·9
50–54 years	82 (49–114)	37% (25–46)	1/2·7	11 (6–25)	5% (3–10)	1/19·8
55–59 years	86 (52–116)	44% (30–54)	1/2·3	14 (7–31)	7% (4–14)	1/14·2
60–64 years	86 (53–114)	52% (36–63)	1/1·9	13 (7–30)	8% (5–17)	1/12·3
65–69 years	84 (53–111)	60% (42–71)	1/1·7	15 (8–33)	10% (6–21)	1/9·7
≥70 years	191 (126–246)	74% (54–86)	1/1·4	35 (19–79)	14% (8–28)	1/7·4
**Males**						
All ages	838 (494–1156)	21% (14–27)	1/4·7	225 (120–509)	6% (3–12)	1/17·4
<20 years	58 (25–84)	4% (2–6)	1/23·1	2 (1–5)	0% (0–0)	1/694·6
20–29 years	66 (34–99)	11% (6–15)	1/9·2	11 (6–25)	2% (1–4)	1/55·8
30–39 years	109 (61–159)	19% (12–25)	1/5·4	26 (14–59)	4% (3–9)	1/22·6
40–49 years	138 (81–194)	28% (18–36)	1/3·5	34 (18–77)	7% (4–14)	1/14·5
50–54 years	81 (49–112)	36% (25–46)	1/2·7	22 (12–51)	10% (6–21)	1/9·9
55–59 years	84 (52–114)	44% (30–54)	1/2·3	27 (14–61)	14% (8–29)	1/7·1
60–64 years	82 (51–109)	52% (36–63)	1/1·9	25 (13–57)	16% (10–33)	1/6·2
65–69 years	77 (49–101)	60% (42–71)	1/1·7	26 (14–59)	21% (12–42)	1/4·9
≥70 years	143 (93–184)	72% (53–85)	1/1·4	52 (28–116)	26% (16–54)	1/3·8

Increased risk is defined as individuals with at least one condition listed in guidelines. High risk is defined as individuals with at least one condition who would require hospitalisation if infected. UI=uncertainty interval. CrI=credible interval.

* For numbers at increased risk, the low estimates were based on a scenario assuming the lower 95% CI values for the age-sex-specific population estimates, disease prevalence rates, and multimorbidity fraction, and assuming *r*=0·7. The high estimates were based on the upper 95% CI values of the same parameters and assume *r*=1·0. For the numbers at high risk, the low estimates were based on a scenario assuming the lower 95% CI values for the age-sex-specific population estimates and lower 95% CrI values published by Verity and colleagues^21^ for infection–hospitalisation ratios in mainland China. The high estimates are based on the higher 95% CI values for the age-sex-specific population estimates and higher 95% CrI values published by Verity and colleagues.^21^

**Figure 2:**
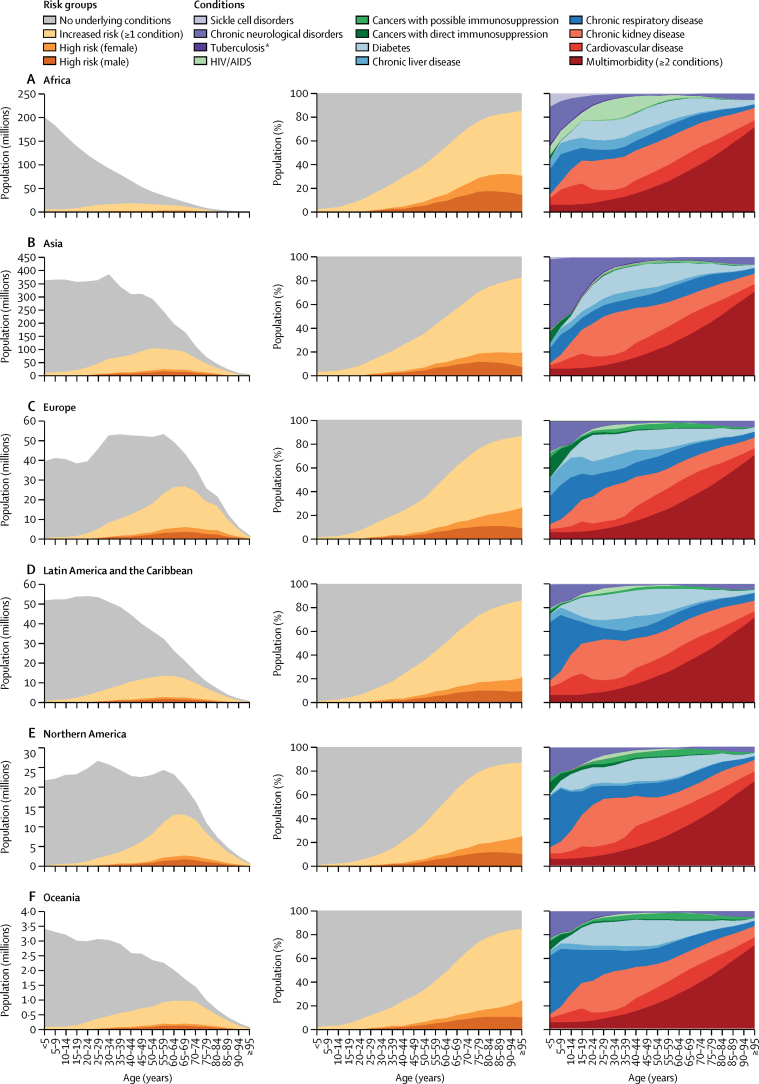
Number and percentage of population at increased risk and high risk of severe COVID-19 by age and region; and distribution of underlying conditions by age and region Each row of graphs presents data for a UN geographical region. The first and second columns show the number of individuals and percentage share of the population, respectively, in each risk group by age, with those at high risk divided into females and males. The third column shows the distribution of the 11 underlying conditions by age, including multimorbidity as a separate category. *Excludes latent infections.

**Figure 3:**
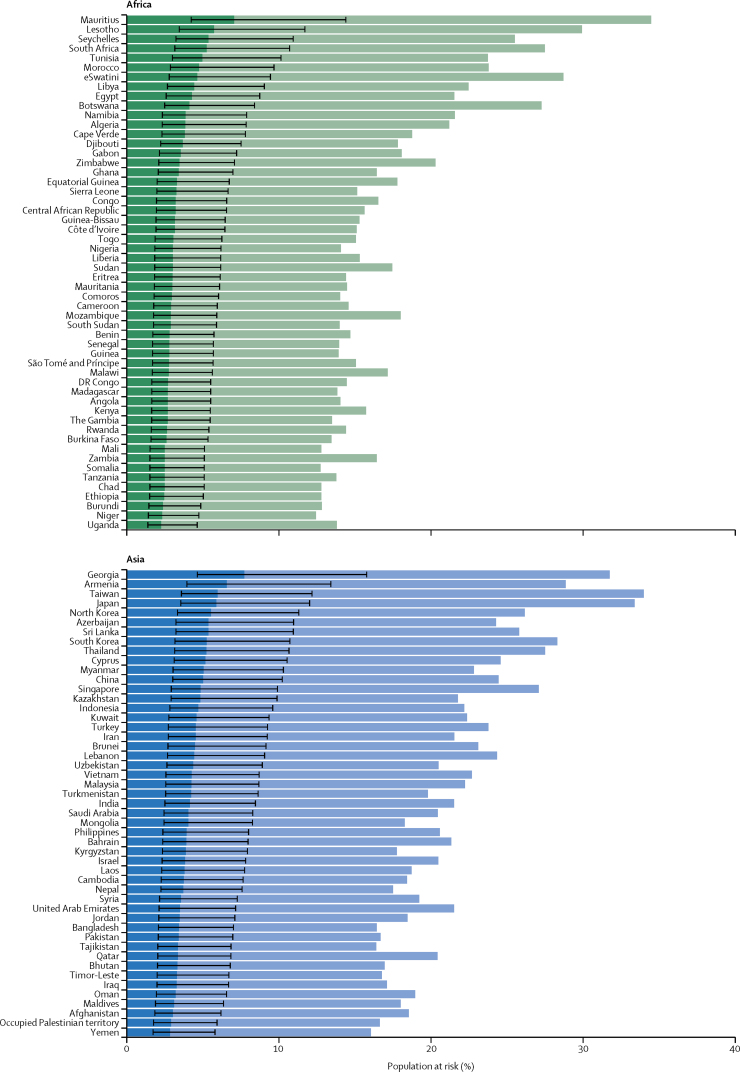

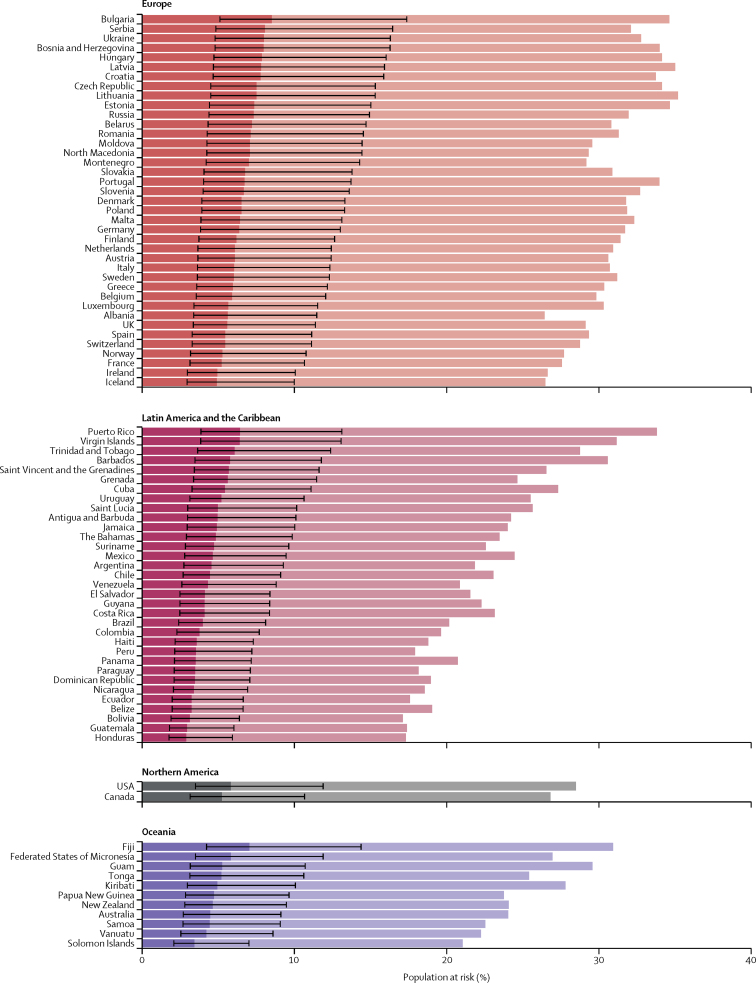
Proportion of population at increased risk and high risk of severe COVID-19 by country and region The total length of each bar represents the share of the population at increased risk (ie, those with at least one condition listed as at increased risk in current guidelines); this excludes individuals considered to be at increased risk by virtue of their age alone. The darker bars represent the share of the population at high risk (ie, those that would require hospital admission if infected), with thin bars representing uncertainty intervals. Here, the population at risk is not age standardised. Thus, differences between countries are driven by differences in the population structure, as well as differences in risk at equivalent ages. This is appropriate when calculating the number and percentage of country populations that might need to be shielded or vaccinated. Another version of this figure shows the age-standardised population at risk (assuming the same population structure in each country), and thus allows more direct comparison of the risk at equivalent ages in different countries (appendix p 16).

**Figure 4:**
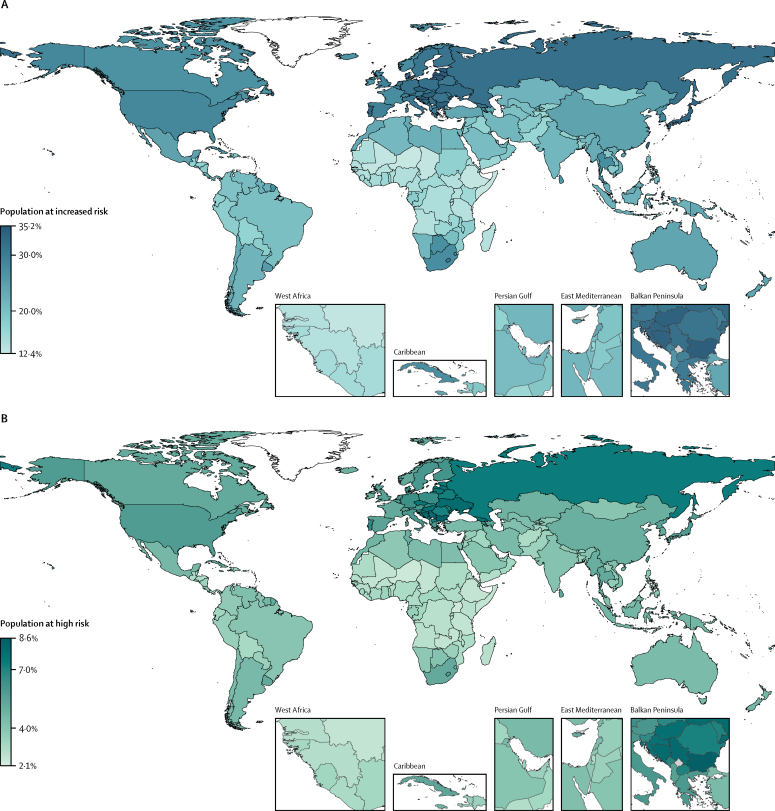
Proportion of population at increased risk and high risk of severe COVID-19 by country For age-standardised estimates, see appendix (p 16).

Based on crude proportions without age standardisation, the share of the population at risk ranged from 16% in Africa to 31% in Europe, consistent with the age profiles of the regions (table 1; Figure 2, Figure 3, Figure 4). The share of the population at increased risk was highest in countries with older populations (eg, Japan, Puerto Rico, and most European countries), African countries with high HIV/AIDS prevalence (eg, eSwatini and Lesotho), and small island nations with high diabetes prevalence (eg, Fiji and Mauritius).

In African countries with high HIV prevalence, excluding the population on ART notably reduced the at-risk proportion: from 29% to 19% in eSwatini, 27% to 20% in Botswana, 30% to 24% in Lesotho, and 27% to 23% in South Africa.

We estimated that 23% (UI 15–29) of the global working age population (15–64 years) have at least one underlying condition. Chronic kidney disease and diabetes were the most common conditions in this age range (figure 2).

Among the 1·7 billion individuals estimated to be at increased risk, we estimated that 0·4 billion (UI 0·2–0·7) individuals—6% (3–8) of the global population—are living with two or more conditions relevant to COVID-19 outcomes (table 1; figure 2; appendix pp 13–14). As expected, this proportion was higher in regions with an older age profile, such as Europe and Northern America.

Our assumption that males were twice as likely to be at high risk than females across all ages means that males represent a larger share of the numbers at high risk; there were approximately twice the number of males at high risk than females in all age groups younger than 65 years, with this ratio becoming less marked in older age groups where males are less represented in the general population (figure 2).

If individuals aged 70 years or older without an underlying condition are considered at risk solely because of their age, then the share of the global population at risk increases from 22% to 24% (UI 18–29; table 1). If all individuals aged 50 years or older without an underlying condition are included, then the share of the global population at risk increases to 34% (UI 30–37; table 1), but this proportion varies considerably by region.

We estimated that 349 million (UI 186–787) people—4% (3–9) of the global population—are at high risk of severe COVID-19 and would require hospital admission if infected (table 2; Figure 2, Figure 3, Figure 4). The proportion of individuals at high risk in each age group ranged from approximately one in every 900 individuals younger than 20 years to one in every five individuals aged 70 years or older (table 2). Age-specific risks for each country are available in the spreadsheet tool, and provide more insight into the actual level of risk within specific age groups. For example, age-specific risks in eSwatini were more than double those in New Zealand in nearly all age groups (age-standardised share of the population at high risk of 8% *vs* 3%; appendix p 16), despite both countries having a very similar share of the population at high risk based on crude percentages of the total population at risk (both 5%; figure 3).

Adjustments for national mix of underlying conditions and age-based frailty were influential in Africa (40% increase in the number at high risk), but less influential in other UN regions (≤11% change in the number at high risk). In Africa, the share of the population at high risk was 2·2% (30 million) without adjustment, 2·7% (36 million) with adjustment for underlying conditions and 3·1% (42 million) with adjustment for both underlying conditions and age-based frailty (appendix p 12). Also, the share of the population at high risk increased from 3·1% (42 million) to 3·7% (49 million) when the RR for HIV was increased from 1·5 to 10·0 (appendix p 12). However, the proportions of the population at increased and high risk estimated for Africa are lower than in other regions, driven by demographics and strong association between severe COVID-19 and age, even after adjusting for underlying conditions and age-based frailty (figure 4). This should not be interpreted as Africa having lower risks of severe COVID-19 disease at equivalent ages than elsewhere, but rather Africa having a lower share of its population living in the oldest (and highest risk) age groups. Indeed, age-standardised rates (assuming each country has the same population structure) show a broadly similar share of the population at risk in most parts of the world, although African countries with high HIV prevalence and small island nations with high prevalence of diabetes still have a high share of the population at risk (appendix p 16).

## Discussion

Based on current guidelines, we estimate that about one in five individuals worldwide has an underlying condition that could put them at increased risk of severe COVID-19 if infected, ranging from less than 5% of those younger than 20 years to more than 66% of those aged 70 years or older. However, for many of these individuals, their condition might not be diagnosed or known to the health system, or their increased risk could be quite modest. Indeed, we estimate that fewer individuals (about one in 20) would actually require hospital admission if infected, ranging from less than 1% of those younger than 20 years to nearly 20% of people aged 70 years or older, rising to more than 25% in males. Whether or not these individuals are actually infected, and whether or not they receive hospital care if their infection is severe, is beyond the scope of this analysis.

Recent estimates from the UN Economic Commission for Africa suggest that an unmitigated pandemic could lead to a substantial proportion of the African continent being infected and 23 million severe cases of COVID-19 requiring hospitalisation.^26^ Our estimates for Africa, based on the same IHRs estimated for mainland China by Verity and colleagues,^21^ were higher (42 million *vs* 23 million), reflecting important adjustments for underlying conditions and age-based frailty. However, even after these adjustments, the total share of the population at high risk is still lower in Africa than in Europe (3·1% *vs* 6·5%). This evidence will need to be carefully communicated to policy makers to avoid complacency about the risk in Africa. First, the lower share of the population at risk simply reflects the much younger populations of countries in Africa compared with Europe, and therefore masks the fact that age-specific risks in African countries tend to be similar or higher than age-specific risks in European countries (appendix p 16). Second, a much higher proportion of severe cases are likely to be fatal in Africa than in Europe, and disruption to health systems could lead to substantial mortality from non-COVID-19 diseases.

If a safe and effective vaccine is produced, then our estimates provide an indication of the volumes that would be required for vaccination of at-risk individuals globally. In the absence of a vaccine, at-risk individuals might need to be shielded by more intensive physical distancing measures than individuals in the wider population. This approach could be especially important at times and places where health systems risk being overwhelmed by cases. At a minimum, timely information should be provided to communities about who is at increased risk according to current guidelines. Simple tools or classifications could also be developed to help individuals to understand their degree of risk on the basis of their individual characteristics.^27^ Improved population-based screening for high-risk conditions could also be considered. Among those who are identified, governments will rely heavily on their adherence to guidelines, such as increased hygiene, physical isolation, and use of home-delivered food and medical care.^6^ Other infection control measures include provision of personal protective equipment and intensive testing of health-care and social care workers in maximum contact with at-risk individuals. Incentives could be introduced to encourage at-risk individuals to reduce or abstain from exposure at workplaces, or relocate to dedicated safe zones.^28^ There is also growing evidence in support of face masks as a means to prevent transmission by those wearing them.^29^ If proven to be effective, or if other measures emerge,^30^ this could also be a practical way of reducing exposure among those who are unable to avoid contact with others, such as daily wage earners or people living with (or caring for) less vulnerable individuals.^31^

Our estimates of the number of individuals at high risk included adjustments for the prevalence and mix of underlying conditions in different countries. This required estimates of the strength of association between each of the 11 underlying conditions and COVID-19 hospital admission. We ran scenarios with different RRs, informed by the few studies that allowed comparison with a control group that was not hospitalised. However, the true strength of association is uncertain and likely to vary across settings. We estimate that a very low proportion of younger individuals (about one in 700 males and one in 1400 females aged <20 years) will develop severe illness if infected. These estimates rely on IHRs from Verity and colleagues,^21^ which assume the same rate of infection in all age groups. However, younger individuals might be less likely to be infected than adults,^10^, ^32^ and consequently could have a higher probability of severe disease on infection than estimated by Verity and colleagues.^33^ In either scenario, the absolute risk of severe disease should be low in younger individuals, but more evidence is needed on the characteristics of younger individuals that develop severe symptoms so they can be identified and shielded effectively.

Our estimates of the number of individuals at high risk in Africa were sensitive to the RR assumed for HIV/AIDS. It is not yet known whether those with HIV are at increased risk of severe disease with COVID-19.^34^ Although it has been shown that widespread introduction of ART reduced the risk of hospitalisation and death associated with seasonal influenza,^35^ a substantial proportion of those on ART remain somewhat immunocompromised.^36^, ^37^ Recent evidence from South Africa has shown that individuals living with HIV have an eight times higher risk of pneumonia hospitalisation associated with seasonal influenza and a three times higher risk of pneumonia death.^38^ Until more evidence emerges, it might be necessary to include individuals with HIV in shielding strategies, irrespective of ART status, with priority given to those not yet receiving treatment.^39^

We included underlying conditions that were listed in any of the guidelines (WHO, the UK, and the USA) and available in GBD 2017. Risk factors included in guidelines but not in GBD (eg, body-mass index [BMI] ≥40 kg/m^2^) were excluded, along with possible risk factors not currently included in guidelines (eg, BMI ≥30 kg/m^2^, ethnicity, and smoking). However, many of these risk factors do not have baseline prevalence data available for 188 countries by age (in 5-year age groups) and sex. Including other risk factors would increase the numbers at increased risk, but there is likely to be substantial overlap with these factors and the underlying conditions already included in the analysis. As our understanding of COVID-19 evolves, guidelines will need to be updated, and baseline prevalence data will need to be improved, particularly on risk factors. Multivariable analyses are emerging on the risk of death among those already admitted to hospital,^24^ but information about the risk of severe disease (ie, requiring hospital admission) among those infected is scarce because very few studies have included patients with COVID-19 who were not admitted to hospital.^23^

We estimated a similar number of males and females to be at increased risk but assumed males were twice as likely to be at high risk. This is consistent with an increasing role of male sex as the severity of COVID-19 increases.^40^ Research in mice infected with severe acute respiratory syndrome coronavirus also found an increased male susceptibility mediated by differences in oestrogen receptor signalling,^41^ while others have noted the concentration of genes involved in the immune system on the X chromosome.^42^ This is clearly a priority for further research.

The association between the prevalence of underlying conditions and other national characteristics, such as economic development, is complex. The prevalence of many of these conditions, except perhaps HIV/AIDS, reflects the epidemiological transition^43^ but survival with these conditions might reflect the performance of the health system.^44^ Hence, it is important to look at the data for each country, which goes beyond what we can report in this Article. Our spreadsheet tool can be used to estimate the number and percentage of country populations targeted under different shielding policies. This allows different health conditions to be included or excluded, different age thresholds to be assessed, and different choices about key assumptions—eg, estimates of the ratio *r* and the multimorbidity fraction by age. The spreadsheet can also be updated with alternative sources of prevalence data if preferred, and specific conditions added or removed as more evidence emerges. A recent analysis from Sweden^45^ provides an opportunity to evaluate our method. By applying our adjustments for clustering to the prevalence data reported in the Swedish study (based on electronic health records), we were able to reproduce the same percentage share of the population at increased risk as that reported in the study.

Our estimates of the share of the population at increased risk are based on prevalence estimates extracted from GBD.^12^ Because GBD produces internally comparable estimates for a comprehensive list of diseases by age, sex, and country, these estimates are well suited to our analysis. GBD prevalence estimates are likely to be higher than prevalence estimated from national databases because they aim to capture cases that might be undiagnosed or not severe enough to be included in electronic health records. For example, more than half of the chronic kidney disease cases included in GBD prevalence estimates represent early-stage disease (stage 1 or 2), which is common and rarely has symptoms.^46^ Several other underlying conditions estimated by GBD are also likely to represent cases that are undiagnosed or not recorded in national databases. In a cross-sectional study in England, more than 20% of diabetes was undiagnosed in all age groups older than 25 years,^47^ and in lower-income settings, this proportion is likely to be much higher.^48^

While our analysis provides numbers of people who could benefit from shielding due to underlying conditions, in practice, the low coverage of diagnosis and treatment for many chronic conditions in low-income settings means that age-based thresholds could play a key role. However, the choice of age threshold needs to be carefully balanced against the proportion of the working age population affected and the adverse mental health consequences that might be associated with long periods of isolation. Our analysis found that around one in five individuals in the working age range had at least one underlying condition relevant to COVID-19 severity. If implemented, shielding of at-risk individuals is likely to be required for several months. This could have a substantial impact on working-age people if they and their household contacts are less economically active for longer than the general population.

We used data from two large studies to adjust for multimorbidity. Both studies could have underestimated the prevalence of some conditions and therefore the extent of multimorbidity, although in Scotland^18^ most of the included conditions were well recorded in routine health care, and in the southern China study,^19^ underlying conditions were well communicated to patients, with information from a community household survey following a standard protocol. However, these studies cannot capture the global diversity of patterns of multimorbidity, which will differ in regions where, for example, there are high prevalences of HIV or sickle cell disorders.

With physical distancing measures of varying intensity in place worldwide, and substantial uncertainty about the transmissibility of the virus in different contexts, the results of any attempt to calculate the number of individuals that will eventually be infected in different countries will be highly uncertain. Nonetheless, we hope our estimates will provide useful a starting point for considering the number of individuals that might need to be shielded or vaccinated as the global COVID-19 pandemic unfolds.
