# Supplementary material for: Global, regional, and national estimates of the population at increased risk of severe COVID-19 due to underlying health conditions in 2020: a modelling study
Source: Lancet Glob Health. 2020 Jun 15;8(8):e1003–17. doi: 10.1016/S2214-109X(20)30264-3 (PMC7295519; doi:10.1016/S2214-109X(20)30264-3)
Supplement: Supplementary appendix [file mmc1.pdf]

THE LANCET
Global Health

Supplementary appendix

This appendix formed part of the original submission and has been peer reviewed. We post it as supplied by the authors.

Supplement to: Clark A, Jit M, Warren-Gash C, et al. Global, regional, and national estimates of the population at increased risk of severe COVID-19 due to underlying health conditions in 2020: a modelling study. *Lancet Glob Health* 2020; published online June 12. http://dx.doi.org/10.1016/S2214-109X(20)30264-3.

Supplementary appendix

Centre for Mathematical Modelling of Infectious Diseases (CMMID) COVID-19 working group 1

Table 1. List of conditions in GBD 2017 with potential to increase the risk of severe COVID-19 illness 2

Methods used to calculate the proportion of individuals with at least one underlying condition 3

Proportion with at least one underlying condition relevant to severe COVID-19 disease 3

Adjustment for multimorbidity 3

Figure 1. Empirical estimates of the ratio r (left panel) and the multimorbidity fraction among those with at least one underlying health condition relevant to COVID-19 (right panel) from cross-sectional studies in Scotland and Southern China 4

Methods for estimating numbers of individuals at high risk 5

Infection hospitalisation ratios 5

Adjustments to IHRs 5

Relative risks for hospital admission 5

Table 2. Summary of evidence on risk factors for COVID-19 hospital admission: used in a scenario to estimate of the number of individuals at high risk of severe COVID disease 7

Sensitivity analysis for estimates of the number of individuals at high risk 11

Table 3. Population in millions (%) at high risk by region: base case and alternative scenarios 12

Table 4. Number of individuals in millions at increased risk of severe COVID-19 illness by age, number of conditions, region, and age threshold: *low scenario estimates* 13

Table 5. Number of individuals in millions at increased risk of severe COVID-19 illness by age, number of conditions, region, and age threshold: *high scenario estimates* 14

Figure 2. Percentage of global population at increased risk of severe COVID-19 disease (left panel) and % change (right panel) when conditions are removed one at a time 15

Figure 3. Alternative version of Figure 3 in main paper showing the age-standardised proportion of population at increased risk and high risk of severe COVID-19 by country and region.. 16

References 17

Centre for Mathematical Modelling of Infectious Diseases (CMMID) COVID-19 working group
Centre for Mathematical Modelling of Infectious Diseases, London School of Hygiene & Tropical Medicine, London, UK

The CMMID COVID-19 working group is (ordered randomly):
Emily S Nightingale, Kathleen O'Reilly, Thibaut Jombart, W John Edmunds, Alicia Rosello, Fiona Yueqian Sun, Katherine E Atkins, Nikos I Bosse, Samuel Clifford, Timothy W Russell, Arminder K Deol, Yang Liu, Simon R Procter, Quentin J Leclerc, Graham Medley, Gwen Knight, James D Munday, Adam J Kucharski, Carl A B Pearson, Petra Klepac, Kiesha Prem, Rein M G J Houben, Akira Endo, Stefan Flasche, Nicholas G Davies, Charlie Diamond, Kevin van Zandvoort, Sebastian Funk, Megan Auzenbergs, Eleanor M Rees, Damien C Tully, Jon C Emery, Billy J Quilty, Sam Abbott, Julian Villabona-Arenas, Stéphane Hué, Joel Hellewell, Amy Gimma, Christopher I Jarvis

Table 1. List of conditions in GBD 2017 with potential to increase the risk of severe COVID-19 illness

#	Category	Causes included in the Global Burden of Disease Study (GBD2017)
1	HIV/AIDS	HIV/AIDS - Drug-susceptible Tuberculosis; HIV/AIDS - Multidrug-resistant Tuberculosis without extensive drug resistance; HIV/AIDS - Extensively drug-resistant Tuberculosis; HIV/AIDS resulting in other diseases
2	Tuberculosis*	Drug-susceptible tuberculosis; Multidrug-resistant tuberculosis without extensive drug resistance; Extensively drug-resistant tuberculosis
3	Cancers with direct immune suppression	Hodgkin lymphoma; Non-Hodgkin lymphoma; Multiple myeloma; Acute lymphoid leukemia; Chronic lymphoid leukemia; Acute myeloid leukemia; Chronic myeloid leukemia; Other leukemia; Other malignant neoplasms; Myelodysplastic, myeloproliferative, and other hematopoietic neoplasms
4	Cancers with possible immune suppression (from treatment therapy)	Lip and oral cavity cancer; Nasopharynx cancer; Other pharynx cancer; Esophageal cancer; Stomach cancer; Colon and rectum cancer; Liver cancer due to hepatitis B; Liver cancer due to hepatitis B; Liver cancer due to hepatitis B; Liver cancer due to hepatitis C; Liver cancer due to alcohol use; Liver cancer due to NASH; Liver cancer due to other causes; Gallbladder and biliary tract cancer; Pancreatic cancer; Larynx cancer; Tracheal, bronchus, and lung cancer; Malignant skin melanoma; Breast cancer; Cervical cancer; Uterine cancer; Ovarian cancer; Prostate cancer; Testicular cancer; Kidney cancer; Bladder cancer; Brain and nervous system cancer; Thyroid cancer; Mesothelioma
5	Cardio-vascular disease	Rheumatic heart disease; Ischemic heart disease; Ischemic stroke; Intracerebral haemorrhage; Subarachnoid haemorrhage; Hypertensive heart disease; Non-rheumatic calcific aortic valve disease; Non-rheumatic degenerative mitral valve disease; Other non-rheumatic valve diseases; Myocarditis; Alcoholic cardiomyopathy; Other cardiomyopathy; Atrial fibrillation and flutter; Aortic aneurysm; Peripheral artery disease; Endocarditis; Other cardiovascular and circulatory diseases; Congenital heart anomalies
6	Chronic respiratory disease	Chronic obstructive pulmonary disease; Silicosis; Asbestosis; Coal workers pneumoconiosis; Other pneumoconiosis; Asthma*; Interstitial lung disease and pulmonary sarcoidosis
7	Chronic liver disease	Cirrhosis and other chronic liver diseases due to hepatitis B; Cirrhosis and other chronic liver diseases due to hepatitis C; Cirrhosis and other chronic liver diseases due to alcohol use; Cirrhosis and other chronic liver diseases due to other causes
8	Diabetes	Diabetes mellitus type 1; Diabetes mellitus type 2
9	Chronic kidney disease	Chronic kidney disease due to diabetes mellitus type 1; Chronic kidney disease due to diabetes mellitus type 2; Chronic kidney disease due to hypertension; Chronic kidney disease due to glomerulonephritis; Chronic kidney disease due to other and unspecified causes
10	Chronic neurological disorders	Alzheimer's disease and other dementias; Parkinson's disease; Multiple sclerosis; Motor neuron disease; Other neurological disorders; Idiopathic developmental intellectual disability; Down syndrome; Neural tube defects
11	Sickle cell disorders	Sickle cell disorders

* we excluded latent tuberculosis and adjusted asthma to better reflect BTS steps 4+.

Methods used to calculate the proportion of individuals with at least one underlying condition
Note: Some of the text from the main paper is repeated here for convenience.

Proportion with at least one underlying condition relevant to severe COVID-19 disease
The GBD study provides prevalence estimates for each disease category separately, but not what we needed, which was the prevalence of people in at least 1 of these categories. Diseases may cluster, for example if they are causally related. To deal with this, we first calculated e, which is the expected proportion of individuals with at least one condition assuming no clustering and that the various prevalences are independent (e.g. the fact that someone has diabetes does not affect their risk of getting cancer) as 1 minus the probability of not having any of the conditions c1, c2, c3….i.e. 1 – (1 – p_c1) x (1 – p_c2) x (1 – p_c3)….

We then estimated the proportion P who have at least one underlying condition as $P = e \times r$, where r is the ratio between the observed and expected percentage of individuals with at least one condition. We based r on evidence from large cross-sectional multimorbidity studies in Scotland[1] and Southern China.[2]

The ratio r was broadly consistent by age, sex and study (see Figure 1 overleaf). For the analysis of both males and females combined, the mean of all age-specific values of r was 0.92 (range 0.86 to 0.99) in Scotland and 0.92 (range (0.75 – 1.15) in China. When extrapolating this value to other countries, we used a ratio of 0.9 for all age groups varied this between 0.7 and 1.0 for transparency. The resulting national estimates of P were constrained to be no less than each country's single most prevalent condition. We conducted sensitivity analyses to explore the impact on results of using the observed age-specific values of r rather than the same value for all ages, but this had a very small impact on the share of the population estimated to be at increased risk i.e. the share of the population at increased risk changed from 22.5% to 22.8%.

Adjustment for multimorbidity
In addition to providing estimates for r, the studies in Scotland and Southern China were also used to calculate the multimorbidity fraction i.e. the proportion of individuals with multiple (two or more) underlying conditions among those with at least one, by age group and sex. All analyses were done using disease categories that matched as closely as possible to the COVID-19-relevant categories defined in our analysis. In both studies this included: CVD (defined as the presence of one or more of coronary heart disease, hypertension, cerebrovascular disease, peripheral arterial disease, heart failure, or atrial fibrillation); chronic neurological disease (defined as one or more of dementia, multiple sclerosis and Parkinson's disease); and CRD (defined as one or both of chronic obstructive pulmonary disease and bronchiectasis). Other COVID-related conditions listed in the main analysis were counted separately. The GBD provide separate estimates for hypertensive heart disease and CKD due to hypertension, but it was not possible to make this distinction in the multimorbidity datasets, so all hypertension was included in the CVD category.

We calculated pooled estimates of the multimorbidity fraction by age and sex and extrapolated these pooled estimates to all countries included in the analysis (see Figure 1 overleaf).

Figure 1. Empirical estimates of the ratio r (left panel) and the multimorbidity fraction among those with at least one underlying health condition relevant to COVID-19 (right panel) from cross-sectional studies in Scotland and Southern China

The top row shows results for females and males combined. The middle row shows results for females only and the bottom row shows results for males only.

The left panel/column shows the ratio between the observed and expected % of individuals with at least one condition by age. Expected estimates were calculated by assuming the prevalences of COVID-19 underling conditions are independent (e.g. the fact that someone has diabetes does not affect their risk of getting cancer) as 1 minus the probability of not having any of the conditions c1, c2, c3….i.e. 1 – (1 – p_c1) x (1 – p_c2) x (1 – p_c3)…. This was then compared to the observed value of the % of individuals with at least one condition based on the same dataset (either Scotland or Southern China). The ratios between expected and observed are shown on the left panel/column below. Both studies indicate that the expected value based on the assumption of independence would provide reasonable estimates of the observed value. In our main analysis we assumed the ratio was 0.9 but varied this between 0.7 and 1.0 in uncertainty analysis.

The right panel/column shows the proportion of those with at least one underlying condition relevant to COVID-19 with multimorbidity (two or more conditions). As expected, this percentage increases with age in both studies. The grey lines represent pooled estimates and 95% confidence intervals based on a 2nd order polynomial model fitted to all data points. Pooled estimates were extrapolated to all countries included in the analysis by age and sex. The lower and upper CI values were used in our low and high estimates in the main paper.

Empirical estimates of the ratio r by age **Empirical estimates of multimorbidity by age**

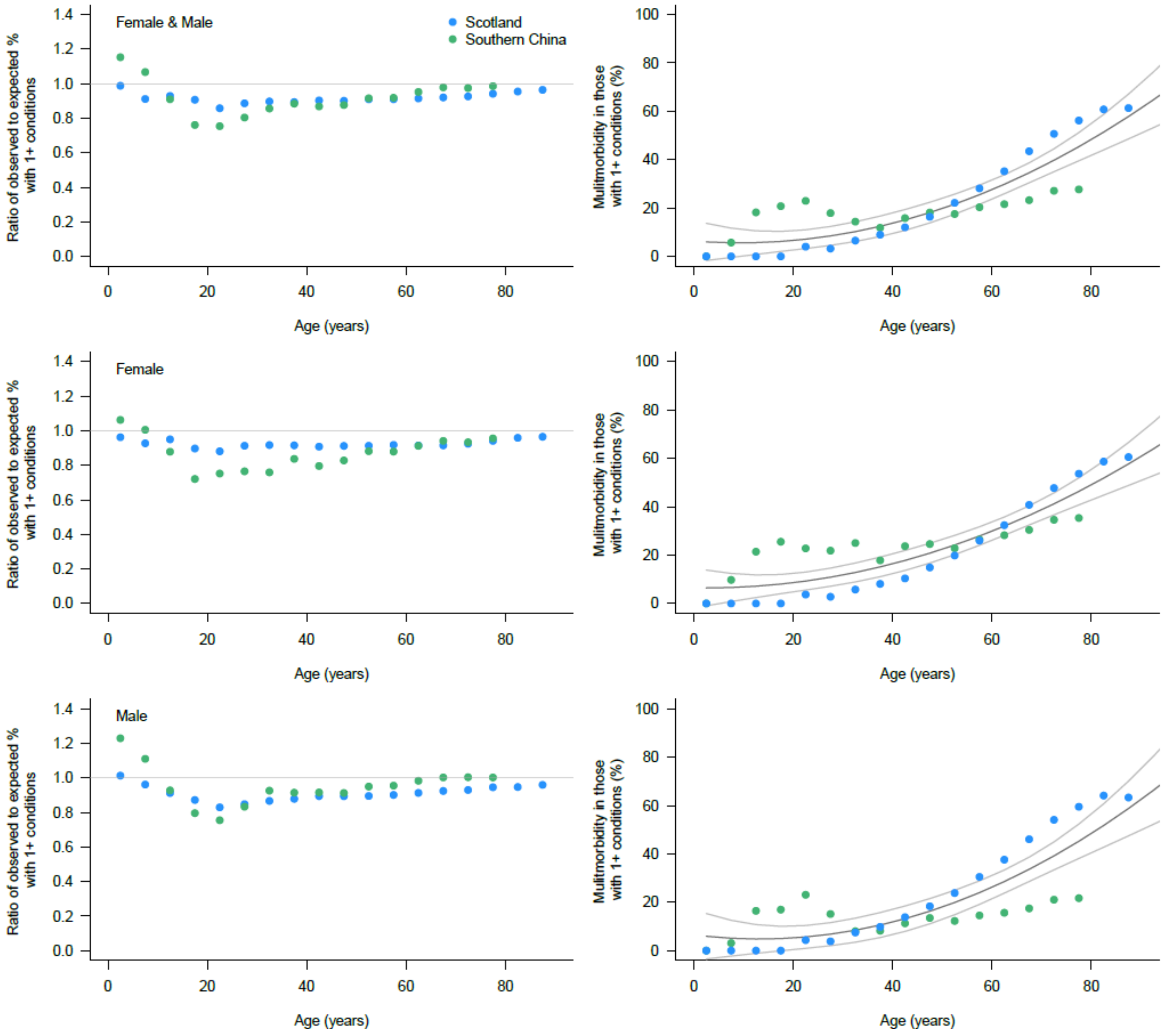

Methods for estimating numbers of individuals at high risk

Infection hospitalisation ratios
To estimate the number of individuals at high risk (those that would require hospital admission if infected) we applied country-level UN estimates of the number of individuals alive in each 5-year age group[3] to age-specific infection hospitalisation ratios (IHRs) recently estimated for mainland China by Verity *et al.*[4] IHRs represent the proportion of people who are infected that would have symptoms severe enough to require hospital admission. The term 'require hospital admission' is consistent with the WHO definition for severe cases.[5] Whether or not these individuals actually receive hospital care will depend on the health system in the country concerned but is beyond the scope of this analysis.

Adjustments to IHRs
We made two adjustments to account for differences between IHRs in China and other countries. The first was designed to capture the effect on IHRs of national variations in prevalence mix. The second was to adjust for infections in given age groups being more severe in higher mortality settings.

1. *Adjusting for underlying conditions*. For each 5-year age group and sex, the prevalence rates for each underlying condition were multiplied by their respective relative risks (RRs) for hospitalisation. RRs of 3.0 were assumed for CKD, diabetes and CVD and of 1.5 for the eight other conditions. The RRs we used were informed by a rapid review of what is currently known about the strength of association between different variables and COVID-19 hospital admission (see table 2 below). The totals were then summed across all 11 conditions and added to the proportion of individuals without underlying conditions, to create a risk score for each 5-year age group. IHRs were then adjusted to account for the ratio of the risk score for the country of interest and China. For example, for males aged 55-59 years the risk score was 2.63 in Afghanistan and 1.95 in China, so the IHR for this age group was multiplied by a ratio of 1.35 (2.63/1.95). This adjustment assumes that for each underlying condition, the RR of hospital admission is the same for every country.

2. *Adjusting for age-based frailty*. Direct interpretation of the first adjustment relies on constant RRs of admission for each condition across countries. To address the likelihood of more severe infections at given ages in higher mortality settings we went further. For each 5-year age group and sex, we divided UN estimates of age-specific life expectancy[3] for China by the equivalent estimate for the country of interest. This ratio (proxy for difference in age-based frailty) was then applied to the IHR for same age group. For example, the life expectancy for males at age 55 years is 22.8 and 19.1 years in China and Afghanistan, respectively, so the IHR for this age group was multiplied by a ratio of 1.19 (22.8/19.1) to generate the estimate for Afghanistan. Country-specific differences in life expectancy may be more extreme than country-specific differences in infection severity because life expectancy depend not only general health status, but also on access to effective health care. Also, the extent to which infections will be more severe in higher mortality settings is still very uncertain. We therefore show results with and without this adjustment.

Thus, for males aged 55-59 years in Afghanistan, separate adjustments for underlying conditions and age-based frailty had the effect of increasing the IHR by 1.61 (IHR x 1.35 x 1.19), from 9% to 15%.

Relative risks for hospital admission
We searched PubMed ("Risk factors" AND "COVID-19") without language restrictions, from database inception until April 5, 2020, and identified 62 studies published between Feb 15, 2020 and March 20, 2020. We later ran a separate PubMed search ("Multivariable" AND "COVID-19") without language restrictions, from database inception until May 25, 2020.

Several studies are emerging on the risk of mortality among those already hospitalised[6,7] but we restricted our analysis of RRs to studies that allowed comparison of hospitalised and non-hospitalised COVID-19 cases. This was because our analysis focused on the risk of severe disease (requiring hospital admission) rather than the risk of death. Three studies from the USA met these criteria. The first contained descriptive data on 6,637 COVID-19 cases reported to the CDC as of 28th March, 2020. For this study, we derived crude univariable RRs for each condition from the reported case counts.[8] The second was a multivariable analysis of 4,103 COVID-19 cases in New York City, between 1st March 2020 and 2nd April 2020.[9] The third was a multivariable study from Northern

California with 1,052 confirmed cases of COVID-19 captured between 1st January and 8th April, 2020.[10] For conditions where evidence was weak or missing, we included studies that did not meet our initial inclusion criteria, either because they were not yet published (i.e. multivariable analyses in Italy[11] and the UK[12]) or because many of the COVID-19 cases in the less severe group were hospitalised i.e. one published meta-analysis of 4 studies from China.[13] Using the evidence from these studies, we summarised the strength of the association with hospital admission (low, moderate, high) and graded our confidence in the strength of that association (low, moderate high).

This rapid review aimed to summarise what is currently known about the strength of association between different variables and COVID-19 hospital admission, but this is unlikely to be exhaustive. We therefore chose to use a very simple stratification of risk for the 11 conditions (either RR= 3 or RR = 1.5) based on the range of RRs reported for these conditions. For three conditions (CKD, diabetes and CVD) we had moderate confidence that the strength of the association would be high based on univariable and multivariable analysis, so assumed a RR of 3.0. For the eight other conditions we assumed a RR of 1.5 because there was insufficient evidence of a significant independent association with hospital admission from multivariable analyses.

RR values for each condition were varied one at a time (assuming low and high values of 1 and 10 respectively) to assess the impact of these changes on the total population at high risk (p 11).

Table 2. Summary of evidence on risk factors for COVID-19 hospital admission: used in a scenario to estimate of the number of individuals at high risk of severe COVID disease

Variable	What is known about risk factors for COVID-19 hospital admission? *(Strength of association is based on evidence restricted to studies that include a control group of COVID-19 patients that were not severe enough to be admitted to hospital)*	Strength of association with admission *(low, moderate, high)*	Confidence about strength of association *(low, moderate, high)*
**Underlying conditions listed in guidelines with available data by age, sex and country**			
Cardiovascular disease	Crude RR = 5.39 (95% CI 4.63 – 6.27) based on 25% vs 5% prevalence with and without admission in the USA (n=6637).[8] Heart failure was also a significant independent predictor (OR = 4.29 [1-89-11.18]) in multivariable analysis in New York City (n=4103).[9] Congestive heart failure was also associated with increased odds of hospital admission (OR = 3.3) based on multivariable analysis from Northern California (n=1,052).[10]	High	Moderate
Chronic kidney disease	Crude RR = 10.19 (95% CI 7.46 – 13.93) based on 10% vs 1% prevalence with and without admission in the USA (n=6637).[8] CKD was also a significant independent predictor (OR =3.07 [1-78-5.52]) in multivariable analysis in New York City (n=4103).[9]	High	Moderate
Diabetes	Crude RR = 4.15 (95% CI 3.63 – 4.74) based on 27% vs 6% prevalence with and without admission in the USA (n=6637).[8] Diabetes was also a significant independent predictor (OR = 2.81 [2.14-3.72]) in multivariable analysis in New York City (n=4103).[9] Type 2 diabetes was also associated with increased odds of hospital admission (OR = 2.2) based on multivariable analysis from Northern California (n=1,052).[10]	High	Moderate
Neurological disorders	Crude RR = 6.48 (95% CI 3.61 – 11.64) based on 2% vs 0% prevalence with and without admission in the USA (n=6637), but the number with neurological disorders was small in this study (n=49),[8] and results on the association with hospital admission were not reported in the multivariable analyses. Data from an unpublished multivariable analysis in Italy (n=2143) found a non-significant association between dementia and hospital admission a (HR = 1.2 [95% CI 0.9-1.7]).[11]	Moderate	Low
Chronic respiratory diseases	Crude RR = 2.33 (95% CI 2.01 – 2.71) based on 16% vs 7% prevalence with and without admission in the USA (n=6637).[8] Not a significant independent predictor (OR = 1.33 [0.96-1.84]) in multivariable analysis in New York City (n=4103).[9] However, both studies included all severities of 'asthma' which is likely to deflate the true odds associated with other chronic respiratory diseases. A meta-analysis of 4 studies from China estimated a pooled univariable OR of 2.46 (95% CI 1.76 – 3.44, I-squared = 0.0%, p = 0.611) but included many non-severe cases that were hospitalised.[14] Data from an unpublished multivariable analysis in Italy (n=2143) found an association between chronic obstructive pulmonary disease (COPD) and hospital admission (HR = 1.9 [95% CI 1.4-2.5]).[11]	Moderate	Low
Tuberculosis (active)	Not data is available, so we assume the same RR assumed for chronic respiratory diseases.	Insufficient data	Low
Chronic liver disease	Crude RR = 2.29 (95% CI 1.22 – 4.31) based on 1% vs 0% prevalence with and without admission in the USA (n=6637), but the number with chronic liver disease was small in this study (n=40).[8] An unpublished study from South Korea found	Moderate	Low
Cancers with possible immunosuppres sion	Crude RR = 2.54 (95% CI 1.98 – 3.25) based on 7% vs 3% prevalence with and without admission in the USA (n=6637) based on a ‘immunosuppressed conditions’ category.[8] Not a significant independent predictor (OR = 1.24 [0.81-1.93]) in multivariable analysis in New York City (n=4103) based on a ‘malignancy’ category.[9] Data from unpublished multivariable analysis in Italy (n=2143) found an association between cancer and hospital admission (HR = 1.4 [95% CI 1.1-1.7]).[11]	Moderate	Low

Variable	What is known about risk factors for COVID-19 hospital admission?<br>*(Strength of association is based on evidence restricted to studies that include a control group of COVID-19 patients that were not severe enough to be admitted to hospital)*	Strength of association with admission<br>*(low, moderate, high)*	Confidence about strength of association<br>*(low, moderate, high)*
Cancers with direct immunosuppression	Crude RR = 2.54 (95% CI 1.98 – 3.25) based on 7% vs 3% prevalence with and without admission in the USA (n=6637) based on a generic category of immunosuppressed conditions.[8] Data from an unpublished multivariable analysis in Italy (n=2143) found an association between cancer and hospital admission (HR = 1.4 [95% CI 1.1-1.7]).[11]	Moderate	Low
HIV / AIDS	Crude RR = 2.54 (95% CI 1.98 – 3.25) based on 7% vs 3% prevalence with and without admission in the USA (n=6637) based on a generic category of immunosuppressed conditions.[8]	Moderate	Low
Sickle cell disorders	Crude RR = 2.54 (95% CI 1.98 – 3.25) based on 7% vs 3% prevalence with and without admission in the USA (n=6637) based on a generic category of immunosuppressed conditions.[8]	Moderate	Low
**Important variables with available data in 188 countries**			
Age	Current guidelines consider those aged 60+ (WHO), 65+ (US) and 70+ years (UK) to be at increased risk. Based on multivariable analysis in New York City (n=4103), ORs were 2.57 (2.06 - 3.20) aged 45-54yrs, 4.17 (3.35-5.20) aged 55-64yrs, 10.91 aged 65-74yrs and 66.79 (44-73-102.62) aged 75+ years, compared to the reference group aged 19-44 years.[9] In this study the median age was 62 vs 41 years with and without admission.	High (reflected in GBD prevalence data and IHRs, which both have a strong age effect)	High
Male gender	Gender is not included in current guidelines. In one multivariable analysis in New York City (n=4103) male gender was a significant independent predictor of hospitalisation (OR = 2.80, 95% CI 2.38 -3.30, p <0.001).[9] Males were twice as likely to be admitted to hospital as females (OR = 1.9, p =0.001) based on multivariable analysis from Northern California (n=1,052).[10] In a large study of hospitalised patients in the UK, 60% of hospitalised patients were male and this effect was seen across all ages (n=16,749).[6] Males had a RR of 1.64 (95% CI 1.25 – 2.14, p <0.001) in a multivariable study that compared a cohort that did and did not have a test-positive hospital admission in the UK (n=428,225).[12] Data from an unpublished multivariable analysis in Italy (n=1866) found an association between male gender and hospital admission (HR = 1.4 [95% CI 1.2-1.6]).[11]	High (for simplicity we assumed males were twice as likely as females to be at high risk i.e. to require hospital admission if infected).	High
**Other possible variables not included in this analysis**			
Pregnancy	The prevalence of pregnancy was the same among COVID-19 hospital admissions (6%) and the wider community in a large study in the UK (n=16749),[6] but this is likely to remain in current guidelines until more evidence emerges e.g. on the risks at different stages of pregnancy.	Low	Low
Hypertension (excluding hypertensive heart disease and CKD caused by hypertension)	GBD includes hypertensive heart disease within CVD and CKD due to hypertension within CKD. Other forms of hypertension are not included. Hypertension unrelated to other underlying conditions is not included in current guidelines, and it is unclear if prevalence data are available for 188 countries by age and sex. In one multivariable analysis in New York City (n=4103), hypertension was not identified as a significant independent predictor (OR = 1.23, 95% CI [0.97 – 1.57], p=0.094).[9] Individuals taking blood pressure medication had a RR of 1.40 (95% CI 1.08 – 1.82, p = 0.01) in a multivariable study that compared a cohort that did and did not have a test-positive hospital admission in the UK (n=428,225).[12] A meta-analysis of 4 studies from China estimated a pooled univariable OR of 2.36 (95% CI 1.46 – 3.83, I-squared = 39.3%, p = 0.176)	Low	Moderate

Variable	What is known about risk factors for COVID-19 hospital admission? *(Strength of association is based on evidence restricted to studies that include a control group of COVID-19 patients that were not severe enough to be admitted to hospital)*	Strength of association with admission *(low, moderate, high)*	Confidence about strength of association *(low, moderate, high)*
	but included many non-severe cases that were hospitalised.[14] Data from an unpublished multivariable analysis in Italy (n=2143) found an association between hypertension and hospital admission (HR = 1.4 [95% CI 1.2-1.6]).[11]		
Current or former smoker	Included in WHO and US guidelines but not UK guidelines. Not a significant independent predictor (OR = 0.71, 95% CI 0.57-0.87, p=0.001) in multivariable analysis in New York City (n=4103).[9] However, current and former smokers were combined into a single category and former smokers appear to have a higher risk. For example, the crude RR for former smokers was 3.36 (95% CI 2.47 – 4.56) based on 5% vs 2% prevalence with and without admission in the USA (n=6637) and ex-smokers were found to have worse survival after hospital admission in a large multivariable analysis in the UK (n=17,425,445) - the hazard ratio was 1.25 (95% CI 1.18-1.33) compared to those that never smoked.[7] It is unclear whether prevalence data would be available for this category by 5-year age group, sex and country (n=188).	Moderate	Low
Ethnicity	Not included in current guidelines. Unclear if prevalence data are available for 188 countries by sex. In one multivariable analysis in New York City (n=4103), other/multiracial race was identified as a significant independent predictor (OR = 1.99, 95% CI [1.62 – 2.45], p<0.001) compared to white race, but African American and Asian race was not a significant predictor.[9] The risk of hospital admission for African Americans was more than double that of non-Hispanic whites (OR = 2.7, p = 0.007) in a multivariable study from Northern California (n=1,052).[10]	Moderate	Moderate
BMI 30-39	Not included in current guidelines. Significant independent predictor (OR = 4.26, 95% CI 3.50-5.20, p<0.001) in multivariable analysis in New York City (n=4103).[9] BMI of 30+ had a RR of 1.97 (95% CI 1.46 – 2.65, p <0.0001) in a multivariable study that compared a cohort that did and did not have a test-positive hospital admission in the UK (n=428,225).[12] Data from an unpublished multivariable analysis in Italy (n=2143) reported a HR = 1.4 [95% CI 0.9-2.0]).[11] Prevalence data are available for adults aged 18+ years, but it is currently unclear if these are available for BMI 30+ by sex and 5-year age group.	High	Moderate
BMI 40+	Included in US/UK guidelines but not WHO guidelines. Significant independent predictor (OR = 6.20, 95% CI 4.21-9.25, p<0.001) in multivariable analysis in New York City (n=4103).[9] Prevalence data are available for adults aged 18+ years, but it is currently unclear if these are available for BMI 40+ by sex and 5-year age group.	High	Moderate
Organ donor recipients	Included in current guidelines but unclear if prevalence data are available for 188 countries by 5-year age and sex. No studies have assessed the association with hospital admission, but it was found to be an important predictor of survival after hospital admission in a large multivariable analysis in the UK (n=17,425,445) - the hazard ratio was 4.27 (95% CI 3.20-5.70).[7]	Insufficient data	Insufficient data
Laboratory markers	Not included in current guidelines. Insufficient data on the prevalence of markers in the general community (e.g. C reactive protein >200 mg/L, d-dimer >500 ng/mL, etc.) so insufficient evidence on risk of hospitalisation. Vitamin D status is emerging as a priority for research and may provide one explanation for the disproportionate number of Black, Asian and Minority Ethnic groups (BAME) experiencing severe symptoms in northern latitudes.[15]	Insufficient data	Insufficient data

Variable	What is known about risk factors for COVID-19 hospital admission? *(Strength of association is based on evidence restricted to studies that include a control group of COVID-19 patients that were not severe enough to be admitted to hospital)*	Strength of association with admission *(low, moderate, high)*	Confidence about strength of association *(low, moderate, high)*
Child malnutrition	Not included in current guidelines. Prevalence data are available but there is insufficient evidence of risk associated with hospital admission.	Insufficient data	Insufficient data
Malaria	Not included in current guidelines. Prevalence data are available but there is insufficient evidence of risk associated with hospital admission.	Insufficient data	Insufficient data
Deprivation	Not included in current guidelines. Unclear if prevalence data are available for 188 countries. Insufficient evidence of risk associated with hospital admission, but was found to be an important predictor of survival after hospital admission in a large multivariable analysis in the UK (n=17,425,445) - the hazard ratio was 1.75 (95% CI 1.60-1.91) in the most deprived quintile compared to the least deprived.[7]	Insufficient data	Insufficient data
Crowded housing	Not included in current guidelines. Unclear if prevalence data are available for 188 countries by age and sex. Insufficient evidence of risk associated with hospital admission.	Insufficient data	Insufficient data
Health and social care workers	Not included in current guidelines. Unclear if prevalence data are available for 188 countries by age and sex. Likely to be an important target group for a future vaccine due to increased risk of transmission.	Insufficient data	Insufficient data
Residents of care homes and other facilities	Included in some guidelines. Unclear if prevalence data are available for 188 countries. Residential care homes are less prevalent in lower income settings but are likely to be an important target group for a future vaccine in high income settings due to increased risk of transmission.	Insufficient data	Insufficient data

Sensitivity analysis for estimates of the number of individuals at high risk

Table 3 (overleaf) summarises the share of the population estimated to be at high risk (those that would require hospital admission if infected) for the base case scenario and for a range of alternative scenarios.

The following assumptions were varied:

1. *Low and high credible intervals of IHRs* – scenarios based on the low and high credible interval values of the IHRs reported in Verity *et al*,[16] were influential. The global population at high risk (4.5%) decreased to 2.7% with low IHRs and increased to 9.1% with high IHRs;

2. *Adjustments for underlying conditions* – removing this adjustment reduced the global population at high risk from 4.5% to 4.0% (and from 3.1% to 2.7% in Africa). In some countries removing this adjustment had a more substantial impact, due to very high prevalence of specific conditions relative to the same conditions in China e.g. diabetes in Fiji, HIV/AIDs in Swaziland;

3. *Adjustments for age-based frailty* - as expected, removing the age-based frailty adjustment resulted in a lower population at high risk in Africa (from 3.1% to 2.6%) and a higher population at high risk in Europe and other high-income settings;

4. *Altering the maximum proportion of the population infected* – our central estimates of the numbers at high risk of severe COVID-19 disease assume there is no theoretical maximum to the proportion of the population that could ever be infected. Thus, the total number of individuals at high risk is calculated by multiplying the total population in each age group by the IHR for the same age group. However, as our understanding of COVID-19 transmission dynamics improves, empirical data may begin to emerge on the scale and duration of immunity acquired from natural infections, and on the proportion of the population that could ever be infected given widespread community transmission. If this is lower than our current assumption of 100%, then fewer individuals would be at high risk using our method. When we varied this value, the global population at high risk decreased from 4.5% assuming all individuals could ever be infected to 4.0%, 3.6%, 3.1% and 2.7% when assuming this value could not exceed 90%, 80%, 70% and 60%, respectively; and,

5. *Altering the RRs for specific conditions* – changing the RR values for each condition one at a time (assuming low and high values of 1 and 10 respectively) had a limited impact on the total population at high risk (<5% increase/decrease). Results were most sensitive to CVD, CKD, diabetes and liver disease (due to its higher prevalence in China relative to many other settings). Increasing the RR for HIV/AIDS from 1.5 to 10.0 was influential in Africa and increased the share of the population at high risk from 3.1% (42 million) to 3.7% (49 million).

Table 3. Population in millions (%) at high risk by region: base case and alternative scenarios

	Africa	Asia	Europe	Latin America and the Caribbean	Northern America	Oceania	Global
**Central, low and high estimates for main analysis**							
Base case scenario<br>• IHRs based on Verity *et al*[16]<br>• IHRs adjusted for underlying conditions<br>• IHRs adjusted for age-based frailty<br>• Maximum % of population infected = 100%<br>• RR = 3.0 (CVD, CKD and diabetes)<br>• RR = 1.5 (all other conditions)	**42 (3.1)**	**208 (4.5)**	**49 (6.5)**	**27 (4.1)**	**21 (5.8)**	**2 (4.6)**	**349 (4.5)**
IHRs based on lower 95% credible intervals	25 (1.9)	124 (2.7)	29 (3.9)	16 (2.4)	13 (3.4)	1 (2.7)	208 (2.7)
IHRs based on upper 95% credible intervals	86 (6.4)	425 (9.2)	99 (13.3)	55 (8.4)	43 (11.8)	4 (9.4)	712 (9.1)
**Scenarios for different IHR adjustments**							
Removing adjustment for age-based frailty	35 (2.6)	202 (4.4)	53 (7.1)	29 (4.4)	25 (6.8)	2 (5.1)	346 (4.4)
Removing adjustment for underlying conditions	36 (2.7)	193 (4.2)	42 (5.6)	24 (3.7)	17 (4.7)	2 (4.1)	315 (4.0)
Removing both adjustments	30 (2.2)	188 (4.1)	46 (6.1)	26 (4.0)	20 (5.5)	2 (4.6)	312 (4.0)
**Scenarios with maximum % of population infected**							
Assumes population infected cannot exceed 90%	38 (2.8)	187 (4.0)	44 (5.9)	24 (3.7)	19 (5.2)	2 (4.1)	314 (4.0)
Assumes population infected cannot exceed 80%	34 (2.5)	166 (3.6)	39 (5.2)	21 (3.3)	17 (4.6)	2 (3.7)	279 (3.6)
Assumes population infected cannot exceed 70%	29 (2.2)	146 (3.1)	34 (4.6)	19 (2.9)	15 (4.0)	1 (3.2)	244 (3.1)
Assumes population infected cannot exceed 60%	25 (1.9)	125 (2.7)	29 (3.9)	16 (2.5)	13 (3.5)	1 (2.8)	209 (2.7)
**Scenarios assuming RR = 1.0 for specific conditions**							
Cardiovascular disease, RR = 1.0	43 (3.2)	211 (4.5)	49 (6.5)	27 (4.2)	21 (5.7)	2 (4.7)	352 (4.5)
Chronic kidney disease, RR = 1.0	40 (3.0)	203 (4.4)	48 (6.4)	26 (4.0)	21 (5.7)	2 (4.5)	340 (4.4)
Chronic respiratory disease, RR = 1.0	42 (3.2)	208 (4.5)	49 (6.5)	27 (4.1)	21 (5.8)	2 (4.6)	349 (4.5)
Chronic liver disease, RR = 1.0	42 (3.2)	209 (4.5)	49 (6.5)	27 (4.1)	21 (5.8)	2 (4.6)	350 (4.5)
Diabetes, RR = 1.0	41 (3.1)	204 (4.4)	48 (6.4)	26 (4.0)	21 (5.6)	2 (4.4)	342 (4.4)
Cancers with direct immunosuppression, RR = 1.0	42 (3.1)	208 (4.5)	49 (6.5)	27 (4.1)	21 (5.8)	2 (4.6)	349 (4.5)
Cancers with possible immunosuppression, RR = 1.0	42 (3.1)	208 (4.5)	48 (6.5)	27 (4.1)	21 (5.8)	2 (4.6)	349 (4.5)
HIV/AIDS, RR = 1.0	42 (3.1)	208 (4.5)	49 (6.5)	27 (4.1)	21 (5.8)	2 (4.6)	348 (4.5)
Tuberculosis, RR = 1.0	42 (3.1)	208 (4.5)	49 (6.5)	27 (4.1)	21 (5.8)	2 (4.6)	349 (4.5)
Chronic neurological disorders, RR = 1.0	42 (3.1)	208 (4.5)	49 (6.5)	27 (4.1)	21 (5.8)	2 (4.6)	349 (4.5)
Sickle cell disorders, RR = 1.0	42 (3.1)	208 (4.5)	49 (6.5)	27 (4.1)	21 (5.8)	2 (4.6)	349 (4.5)
**Scenarios assuming RR = 10.0 for specific conditions**							
Cardiovascular disease, RR = 10.0	41 (3.1)	204 (4.4)	49 (6.5)	26 (4.0)	22 (5.9)	2 (4.4)	343 (4.4)
Chronic kidney disease, RR = 10.0	46 (3.4)	218 (4.7)	50 (6.6)	28 (4.3)	22 (5.8)	2 (4.7)	365 (4.7)
Chronic respiratory disease, RR = 10.0	41 (3.0)	211 (4.6)	48 (6.5)	26 (4.0)	21 (5.7)	2 (4.8)	349 (4.5)
Chronic liver disease, RR = 10.0	41 (3.0)	196 (4.2)	47 (6.3)	26 (4.0)	19 (5.2)	2 (4.2)	331 (4.3)
Diabetes, RR = 10.0	44 (3.3)	217 (4.7)	51 (6.8)	28 (4.3)	23 (6.2)	2 (5.0)	366 (4.7)
Cancers with direct immunosuppression, RR = 10.0	41 (3.1)	206 (4.5)	49 (6.5)	26 (4.0)	21 (5.8)	2 (4.7)	346 (4.4)
Cancers with possible immunosuppression, RR = 10.0	41 (3.0)	205 (4.4)	51 (6.8)	27 (4.1)	23 (6.3)	2 (5.0)	348 (4.5)
HIV/AIDS, RR = 10.0	49 (3.7)	209 (4.5)	49 (6.6)	27 (4.2)	22 (5.9)	2 (4.6)	358 (4.6)
Tuberculosis, RR = 10.0	42 (3.1)	209 (4.5)	48 (6.4)	26 (4.1)	21 (5.7)	2 (4.6)	348 (4.5)
Chronic neurological disorders, RR = 10.0	42 (3.1)	213 (4.6)	47 (6.3)	26 (4.0)	20 (5.5)	2 (4.5)	350 (4.5)
Sickle cell disorders, RR = 10.0	42 (3.2)	208 (4.5)	49 (6.5)	27 (4.1)	21 (5.8)	2 (4.6)	349 (4.5)

Table 4. Number of individuals in millions at increased risk of severe COVID-19 illness by age, number of conditions, region, and age threshold: ***low scenario estimates***

For numbers at increased risk, the low estimates were based on a scenario assuming the lower 95% CI values for the age-sex-specific population estimates, disease prevalence rates, and multimorbidity fraction, and assume an r ratio of 0.7.

	Africa (n=1179.0) million	Asia (n=4126.8) million	Europe (n=685.8) million	Latin America and the Caribbean (n=579.8) million	Northern America (n=329.0) million	Oceania (n=38.3) million	Global (n=6938.6) million
**Population by number of conditions**							
**No conditions**							
<15 years	466.7 (40%)	947.5 (23%)	109.0 (16%)	137.3 (24%)	59.0 (18%)	8.9 (23%)	**1728.5 (25%)**
15-49 years	507.9 (43%)	1910.2 (46%)	272.0 (40%)	276.4 (48%)	138.9 (42%)	16.6 (43%)	**3122.1 (45%)**
50-54 years	26.9 (2%)	194.9 (5%)	36.3 (5%)	24.1 (4%)	15.4 (5%)	1.7 (4%)	**299.2 (4%)**
55-59 years	20.2 (2%)	151.7 (4%)	34.0 (5%)	19.9 (3%)	14.6 (4%)	1.5 (4%)	**241.9 (3%)**
60-64 years	14.4 (1%)	112.6 (3%)	28.2 (4%)	14.8 (3%)	12.4 (4%)	1.2 (3%)	**183.6 (3%)**
65-69 years	9.5 (<1%)	87.0 (2%)	22.0 (3%)	10.7 (2%)	9.3 (3%)	0.9 (2%)	**139.4 (2%)**
≥70 years	10.7 (<1%)	108.8 (3%)	40.1 (6%)	15.4 (3%)	15.1 (5%)	1.6 (4%)	**191.7 (3%)**
All ages	1056.3 (90%)	3512.6 (85%)	541.7 (79%)	498.6 (86%)	264.7 (80%)	32.4 (85%)	**5906.2 (85%)**
**One condition only**							
<15 years	8.0 (<1%)	20.8 (<1%)	0.9 (<1%)	1.2 (<1%)	0.5 (<1%)	0.1 (<1%)	**31.4 (<1%)**
15-49 years	58.7 (5%)	214.9 (5%)	28.8 (4%)	26.4 (5%)	11.2 (3%)	1.6 (4%)	**341.5 (5%)**
50-54 years	8.5 (<1%)	52.1 (1%)	9.1 (1%)	6.6 (1%)	4.3 (1%)	0.4 (1%)	**81.0 (1%)**
55-59 years	7.8 (<1%)	49.8 (1%)	11.3 (2%)	6.8 (1%)	5.5 (2%)	0.4 (1%)	**81.6 (1%)**
60-64 years	6.8 (<1%)	44.9 (1%)	12.3 (2%)	6.2 (1%)	6.2 (2%)	0.4 (1%)	**76.9 (1%)**
65-69 years	5.5 (<1%)	40.9 (<1%)	12.1 (2%)	5.4 (<1%)	5.9 (2%)	0.4 (1%)	**70.4 (1%)**
≥70 years	8.1 (<1%)	66.9 (2%)	30.4 (4%)	10.7 (2%)	13.2 (4%)	1.1 (3%)	**130.4 (2%)**
All ages	103.4 (9%)	490.3 (12%)	104.8 (15%)	63.3 (11%)	46.8 (14%)	4.4 (12%)	**813.0 (12%)**
**Multiple (two or more) conditions**							
<15 years	0.1 (<1%)	0.1 (<1%)	0.0 (<1%)	0.0 (<1%)	0.0 (<1%)	0.0 (<1%)	**0.2 (<1%)**
15-49 years	5.1 (<1%)	20.5 (<1%)	3.0 (<1%)	2.6 (<1%)	1.2 (<1%)	0.2 (<1%)	**32.6 (<1%)**
50-54 years	1.8 (<1%)	11.1 (<1%)	1.9 (<1%)	1.4 (<1%)	0.9 (<1%)	0.1 (<1%)	**17.2 (<1%)**
55-59 years	2.2 (<1%)	13.7 (<1%)	3.1 (<1%)	1.9 (<1%)	1.5 (<1%)	0.1 (<1%)	**22.5 (<1%)**
60-64 years	2.4 (<1%)	15.8 (<1%)	4.3 (<1%)	2.2 (<1%)	2.2 (<1%)	0.2 (<1%)	**27.1 (<1%)**
65-69 years	2.4 (<1%)	18.0 (<1%)	5.4 (<1%)	2.4 (<1%)	2.6 (<1%)	0.2 (<1%)	**31.0 (<1%)**
≥70 years	5.2 (<1%)	44.7 (1%)	21.6 (3%)	7.3 (1%)	9.2 (3%)	0.7 (2%)	**88.8 (1%)**
All ages	19.3 (2%)	124.0 (3%)	39.3 (6%)	17.9 (3%)	17.6 (5%)	1.4 (4%)	**219.4 (3%)**
**Population at increased risk of severe COVID-19 disease**							
At least one condition	122.7 (10%)	614.2 (15%)	144.1 (21%)	81.2 (14%)	64.3 (20%)	5.9 (15%)	**1032.4 (15%)**
Older people with no conditions							
≥50 years	81.7 (7%)	654.8 (16%)	160.6 (23%)	84.9 (15%)	66.8 (20%)	6.9 (18%)	**1055.7 (15%)**
≥55 years	54.8 (5%)	460.0 (11%)	124.3 (18%)	60.8 (10%)	51.4 (16%)	5.2 (14%)	**756.5 (11%)**
≥60 years	34.6 (3%)	308.3 (7%)	90.3 (13%)	40.9 (7%)	36.7 (11%)	3.7 (10%)	**514.6 (7%)**
≥65 years	20.2 (2%)	195.8 (5%)	62.1 (9%)	26.1 (5%)	24.4 (7%)	2.5 (7%)	**331.0 (5%)**
≥70 years	10.7 (<1%)	108.8 (3%)	40.1 (6%)	15.4 (3%)	15.1 (5%)	1.6 (4%)	**191.7 (3%)**
People with at least one condition plus older people with no conditions							
≥50 years	204.4 (17%)	1269.1 (31%)	304.7 (44%)	166.1 (29%)	131.1 (40%)	12.8 (33%)	**2088.1 (30%)**
≥55 years	177.5 (15%)	1074.2 (26%)	268.4 (39%)	142.0 (24%)	115.7 (35%)	11.1 (29%)	**1788.9 (26%)**
≥60 years	157.3 (13%)	922.6 (22%)	234.4 (34%)	122.1 (21%)	101.1 (31%)	9.6 (25%)	**1547.0 (22%)**
≥65 years	142.9 (12%)	810.0 (20%)	206.2 (30%)	107.3 (19%)	88.7 (27%)	8.4 (22%)	**1363.4 (20%)**
≥70 years	133.4 (11%)	723.0 (18%)	184.1 (27%)	96.6 (17%)	79.4 (24%)	7.4 (19%)	**1224.1 (18%)**

Table 5. Number of individuals in millions at increased risk of severe COVID-19 illness by age, number of conditions, region, and age threshold: *high scenario estimates*

For numbers at increased risk, the high estimates were based on a scenario assuming the upper 95% CI values for the age-sex-specific population estimates, disease prevalence rates, and multimorbidity fraction, and assume an r ratio of 1.0.

	Africa (n=1498.4) million	Asia (n=5136.0) million	Europe (n=808.0) million	Latin America and the Caribbean (n=724.0) million	Northern America (n=410.2) million	Oceania (n=45.3) million	Global (n=8621.9) million
**Population by number of conditions**							
**No conditions**							
<15 years	576.5 (38%)	1139.6 (22%)	125.6 (16%)	168.9 (23%)	72.4 (18%)	10.4 (23%)	**2093.4 (24%)**
15-49 years	559.8 (37%)	2059.0 (40%)	278.7 (34%)	307.8 (43%)	156.0 (38%)	17.1 (38%)	**3378.4 (39%)**
50-54 years	23.0 (2%)	175.2 (3%)	31.1 (4%)	21.8 (3%)	13.9 (3%)	1.4 (3%)	**266.5 (3%)**
55-59 years	15.7 (1%)	125.7 (2%)	26.3 (3%)	16.5 (2%)	11.8 (3%)	1.2 (3%)	**197.1 (2%)**
60-64 years	9.5 (<1%)	83.1 (2%)	18.5 (2%)	10.7 (1%)	8.4 (2%)	0.8 (2%)	**131.0 (2%)**
65-69 years	5.1 (<1%)	56.0 (1%)	11.7 (1%)	6.5 (<1%)	4.9 (1%)	0.5 (1%)	**84.7 (<1%)**
≥70 years	3.5 (<1%)	47.6 (<1%)	11.7 (1%)	5.7 (<1%)	4.0 (<1%)	0.5 (1%)	**73.0 (<1%)**
All ages	1193.1 (80%)	3686.1 (72%)	503.6 (62%)	537.9 (74%)	271.5 (66%)	32.0 (71%)	**6224.2 (72%)**
**One condition only**							
<15 years	25.5 (2%)	60.0 (1%)	3.8 (<1%)	3.8 (<1%)	1.7 (<1%)	0.3 (<1%)	**95.1 (1%)**
15-49 years	138.0 (9%)	504.8 (10%)	65.2 (8%)	60.6 (8%)	26.6 (6%)	3.7 (8%)	**799.0 (9%)**
50-54 years	17.6 (1%)	107.2 (2%)	18.0 (2%)	13.3 (2%)	8.6 (2%)	0.8 (2%)	**165.5 (2%)**
55-59 years	15.6 (1%)	98.8 (2%)	21.4 (3%)	13.3 (2%)	10.6 (3%)	0.9 (2%)	**160.5 (2%)**
60-64 years	13.3 (<1%)	86.4 (2%)	22.5 (3%)	11.9 (2%)	11.4 (3%)	0.9 (2%)	**146.4 (2%)**
65-69 years	10.2 (<1%)	75.7 (1%)	21.0 (3%)	10.0 (1%)	10.4 (3%)	0.8 (2%)	**128.3 (1%)**
≥70 years	13.0 (<1%)	104.9 (2%)	42.6 (5%)	16.4 (2%)	19.2 (5%)	1.6 (3%)	**197.7 (2%)**
All ages	233.2 (16%)	1037.8 (20%)	194.6 (24%)	129.5 (18%)	88.5 (22%)	8.9 (20%)	**1692.5 (20%)**
**Multiple (two or more) conditions**							
<15 years	3.7 (<1%)	8.7 (<1%)	0.6 (<1%)	0.5 (<1%)	0.2 (<1%)	0.0 (<1%)	**13.8 (<1%)**
15-49 years	27.5 (2%)	106.9 (2%)	14.6 (2%)	13.1 (2%)	6.0 (1%)	0.8 (2%)	**168.9 (2%)**
50-54 years	6.4 (<1%)	38.7 (<1%)	6.5 (<1%)	4.8 (<1%)	3.1 (<1%)	0.3 (<1%)	**59.9 (<1%)**
55-59 years	6.8 (<1%)	42.9 (<1%)	9.3 (1%)	5.8 (<1%)	4.6 (1%)	0.4 (<1%)	**69.8 (<1%)**
60-64 years	7.0 (<1%)	45.6 (<1%)	11.9 (1%)	6.3 (<1%)	6.0 (1%)	0.5 (1%)	**77.3 (<1%)**
65-69 years	6.7 (<1%)	49.5 (<1%)	13.8 (2%)	6.6 (<1%)	6.8 (2%)	0.5 (1%)	**83.9 (<1%)**
≥70 years	13.8 (<1%)	119.7 (2%)	53.2 (7%)	19.6 (3%)	23.4 (6%)	1.9 (4%)	**231.6 (3%)**
All ages	72.0 (5%)	412.1 (8%)	109.8 (14%)	56.7 (8%)	50.2 (12%)	4.4 (10%)	**705.2 (8%)**
**Population at increased risk of severe COVID-19 disease**							
At least one condition	305.2 (20%)	1449.9 (28%)	304.4 (38%)	186.2 (26%)	138.7 (34%)	13.3 (29%)	**2397.7 (28%)**
Older people with no conditions							
≥50 years	56.8 (4%)	487.6 (9%)	99.2 (12%)	61.2 (8%)	43.1 (11%)	4.5 (10%)	**752.4 (9%)**
≥55 years	33.8 (2%)	312.4 (6%)	68.1 (8%)	39.3 (5%)	29.2 (7%)	3.1 (7%)	**485.8 (6%)**
≥60 years	18.1 (1%)	186.7 (4%)	41.8 (5%)	22.8 (3%)	17.3 (4%)	1.9 (4%)	**288.7 (3%)**
≥65 years	8.6 (<1%)	103.6 (2%)	23.4 (3%)	12.1 (2%)	9.0 (2%)	1.0 (2%)	**157.7 (2%)**
≥70 years	3.5 (<1%)	47.6 (<1%)	11.7 (1%)	5.7 (<1%)	4.0 (<1%)	0.5 (1%)	**73.0 (<1%)**
People with at least one condition plus older people with no conditions							
≥50 years	362.1 (24%)	1937.5 (38%)	403.6 (50%)	247.3 (34%)	181.8 (44%)	17.8 (39%)	**3150.1 (37%)**
≥55 years	339.0 (23%)	1762.3 (34%)	372.5 (46%)	225.5 (31%)	167.9 (41%)	16.4 (36%)	**2883.5 (33%)**
≥60 years	323.3 (22%)	1636.6 (32%)	346.3 (43%)	209.0 (29%)	156.1 (38%)	15.2 (33%)	**2686.4 (31%)**
≥65 years	313.8 (21%)	1553.5 (30%)	327.8 (41%)	198.3 (27%)	147.7 (36%)	14.3 (32%)	**2555.4 (30%)**
≥70 years	308.8 (21%)	1497.5 (29%)	316.1 (39%)	191.8 (26%)	142.7 (35%)	13.8 (30%)	**2470.7 (29%)**

Figure 2. Percentage of global population at increased risk of severe COVID-19 disease (left panel) and % change (right panel) when conditions are removed one at a time

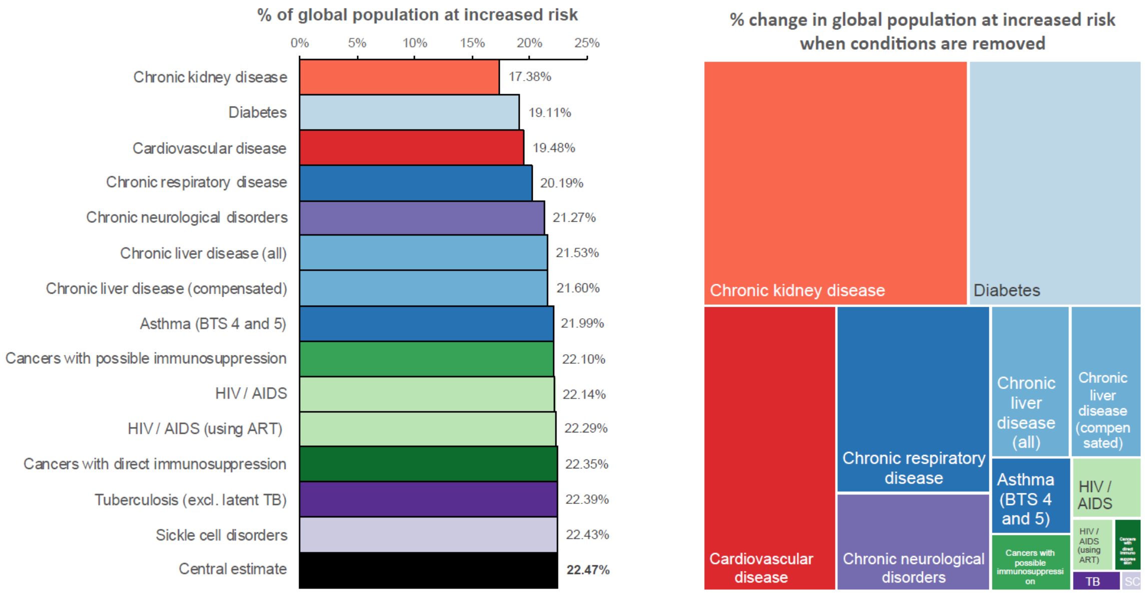

The black shaded bar at the bottom represents the central estimate of the global population that are at increased risk. All other bars above show how this value changes when each of the conditions are removed one at a time. The most influential conditions are at the top of the bar chart and represent larger areas on the map shown on the right side.

Figure 3. Alternative version of Figure 3 in main paper showing the age-standardised proportion of population at increased risk and high risk of severe COVID-19 by country and region

Figure 3 in the main paper shows the share of the population at risk in different countries based on real-world differences in population structure and disease prevalence. This information is important when calculating the numbers that might need to be shielded or vaccinated but does not allow direct comparison of risks at equivalent ages in different countries. In this alternative version (see below), circles have been added to show the age-standardised share of the population at high risk (black circles) and increased risk (open circles). These assume each country has the same WHO standard reference population.[17] A low age-adjusted population at risk in countries with older populations (eg, Japan, Europe and Puerto Rico) helps to confirm that older age is the main reason why these countries have a high unadjusted population at risk. Similarly, a high age-adjusted population at risk in African countries with high HIV prevalence (eg, eSwatini, Lesotho) and small island nations with high diabetes prevalence (eg. Fiji, Mauritius) explains why these countries have a high unadjusted population at risk, despite having younger populations. Differences in demography can mask important differences in age-specific risks that may be revealed by age-standardisation. For example, in eSwatini and New Zealand the population at high risk is 5% in both countries, but when risks are compared for equivalent age groups (within the spreadsheet tool) the age-specific risks in eSwatini are more than double those in New Zealand (consistent with eSwatini having a higher age-adjusted population at high risk ie, 8% vs 3%). Thus, although younger populations will tend to have a lower share of the population at risk than older populations, their risk at equivalent ages could still be higher.

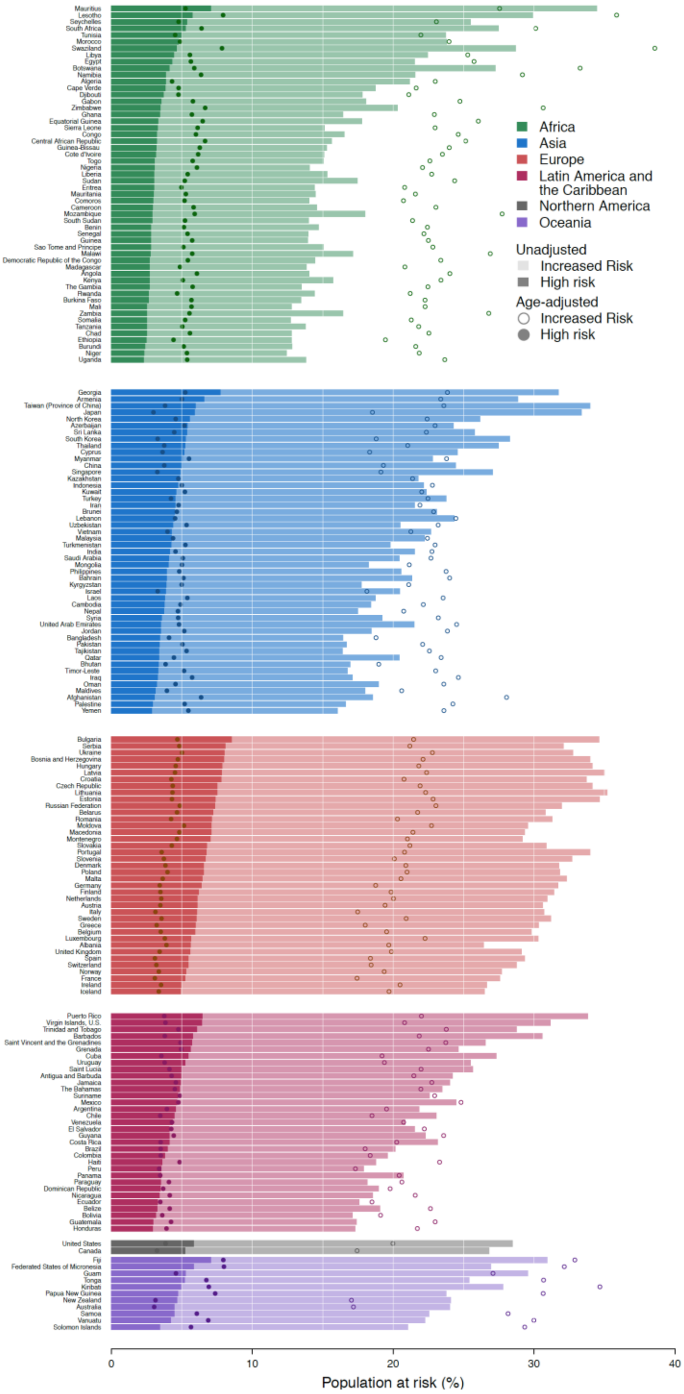

References

1. Barnett K, Mercer SW, Norbury M, Watt G, Wyke S, Guthrie B. Epidemiology of multimorbidity and implications for health care, research, and medical education: a cross-sectional study. *Lancet* 2012; **380**(9836): 37-43.

2. Wang HH, Wang JJ, Wong SY, et al. Epidemiology of multimorbidity in China and implications for the healthcare system: cross-sectional survey among 162,464 community household residents in southern China. *BMC Med* 2014; **12**: 188.

3. Division UNP. UNPOP (2019 Revision) estimates of population by single calendar year (2020), age, sex and country. Available at: https://population.un.org/wpp/Download/Standard/Interpolated/ [Accessed 1st April 2020]. 2020.

4. ECA. COVID-19 in Africa. Protecting lives and economies. Available at: https://www.uneca.org/publications/covid-19-africa-protecting-lives-and-economies. 2020.

5. WHO. Global Surveillance for human infection with coronavirus disease (COVID-19). Available at: https://www.who.int/publications-detail/global-surveillance-for-human-infection-with-novel-coronavirus-(2019-ncov). 2020.

6. Annemarie B Docherty, Ewen M Harrison, Christopher A Green, et al. Features of 16,749 hospitalised UK patients with COVID-19 using the ISARIC WHO Clinical Characterisation Protocol. medRxiv 2020.04.23.20076042; Available at: https://doi.org/10.1101/2020.04.23.20076042. 2020.

7. Elizabeth Williamson, Alex J Walker, Krishnan J Bhaskaran, et al. OpenSAFELY: factors associated with COVID-19-related hospital death in the linked electronic health records of 17 million adult NHS patients. The OpenSAFELY Collaborative, Available at: medRxiv 2020.05.06.20092999; Available at: https://doi.org/10.1101/2020.05.06.20092999. 2020.

8. CDC. Preliminary Estimates of the Prevalence of Selected Underlying Health Conditions Among Patients with Coronavirus Disease 2019 — United States, Centers for Disease Control and Prevention. February 12–March 28, 2020. Available at: https://www.cdc.gov/mmwr/volumes/69/wr/pdfs/mm6913e2-H.pdf?deliveryName=USCDC_921-DM24524. *MMWR* 2020; **Early Release / Vol. 69**.

9. Christopher M. Petrilli, Simon A. Jones, Jie Yang, et al. Factors associated with hospital admission and critical illness among 5279 people with coronavirus disease 2019 in New York City: prospective cohort study. BMJ. 2020;369:m1966. Published 2020 May 22.

10. Kristen M. J. Azar, Zijun Shen, Robert J. Romanelli, et al. Disparities In Outcomes Among COVID-19 Patients In A Large Health Care System In California. https://doi.org/10.1377/hlthaff.2020.00598. *Health Affairs* 2020.

11. Giorgi Rossi P, Marino M, Formisano D, Venturelli F, Vicentini M, Grilli R. Characteristics and outcomes of a cohort of SARS-CoV-2 patients in the Province of Reggio Emilia, Italy. *medRxiv* 2020: 2020.04.13.20063545.

12. Frederick K Ho, Carlos A Celis-Morales, Stuart R Gray, et al. Modifiable and non-modifiable risk factors for COVID-19: results from UK Biobank. medRxiv preprint doi: https://doi.org/10.1101/2020.04.28.20083295. Version posted May 2, 2020. 2020.

13. Jing Yang, Ya Zheng, Xi Gou, et al. Prevalence of comorbidities in the novel Wuhan coronavirus (COVID-19) infection: a systematic review and meta-analysis. Available at: https://www.ncbi.nlm.nih.gov/pmc/articles/PMC7194638/pdf/main.pdf. *International Journal of Infectious Diseases* 2020; **94**: 91-5.

14. Yang J, Zheng Y, Gou X, et al. Prevalence of comorbidities and its effects in patients infected with SARS-CoV-2: a systematic review and meta-analysis. *Int J Infect Dis* 2020; **94**: 91-5.

15. Mitchell F. Vitamin-D and COVID-19: do deficient risk a poorer outcome? Available at: https://doi.org/10.1016/S2213-8587(20)30183-2. *Lancet Diabetes Endocrinol* 2020.

16. Verity R, Okell LC, Dorigatti I, et al. Estimates of the severity of coronavirus disease 2019: a model-based analysis. *Lancet Infect Dis* 2020.

17. Omar B. Ahmed, Boschi-Pinto C, Lopez AD, Murray CJ, Lozano R, Inoue M. Table 4. WHO World Standard Population Distribution (%), based on world average population between 2000-2025. Age standardisation of rates: a new WHO standard. GPE Discussion Paper Series: No.31. EIP/GPE/EBD. World Health Organization 2001. Available at: https://www.who.int/healthinfo/paper31.pdf?ua=1 (accessed June 8 2020). 2001.
